# Repeated ablations of mature Tmem10^+^ oligodendrocytes recapitulates key pathological features of multiple sclerosis with prolonged demyelination

**DOI:** 10.1038/s41419-025-07996-0

**Published:** 2025-10-06

**Authors:** Feiyan Zhu, Haijiao Huang, Yuting Shu, Guoru Ren, Bo Jing, Honglin Tan, Wanxiang Jiang, Yiyuan Cui, Paul F. Worley, Bo Xiao, Mina Chen

**Affiliations:** 1 Departments of Neuroscience and Systems Biology, School of Life Sciences, Southern University of Science and Technology, Shenzhen Key Laboratory for Gene Regulation and Systems Biology, 518000, Shenzhen, People’s Republic of China; 2 Department of Biotherapy, Cancer Center and State Key Laboratory of Biotherapy, West China Hospital, Sichuan University, 610041, Chengdu, PR China; 3 The Solomon H. Snyder Department of Neuroscience, Johns Hopkins University School of Medicine, Baltimore, MD 21205 USA

**Keywords:** Multiple sclerosis, Neuroimmunology, Multiple sclerosis

## Abstract

Managing multiple sclerosis (MS), a chronic, neuroinflammatory and demyelinating disease, remains an unmet medical need. A great obstacle to developing therapeutic interventions altering the disease process is the lack of a suitable disease model recapitulating its core pathologies, especially the process of myelin damage and regeneration against the backdrop of persistent neuroinflammation. Now we report the generation and characterization of a new demyelination/remyelination model with key pathological features of MS through repeated ablations of Tmem10^+^ oligodendrocytes. Using the Tmem10-Cre driver that targets expression of diphtheria toxin receptor (DTR) in Tmem10^+^ (mature myelin-forming) oligodendrocytes, we conducted 3 rounds of Tmem10^+^ oligodendrocyte ablations over the span of 9 weeks in young adult mice by injecting diphtheria toxin (DT). Three rounds of the oligodendrocyte ablation caused prolonged demyelination and axonal injury in the backdrop of chronic neuroinflammation, including gliosis and lymphocyte infiltration. In response to the oligodendrocyte ablations, the proliferation and differentiation of OPCs were accelerated for the regeneration of myelin-forming oligodendrocytes, leading to spontaneous remyelination. This de-/re-myelination model presents a wide “time window” between the onset of demyelination and spontaneous remyelination, which makes it suitable for assessing the efficacy of therapeutic pro-remyelinating agents, as demonstrated by administering Benztropine to this demyelination model.

## Introduction

Multiple sclerosis (MS) is a chronic and debilitating inflammatory, demyelinating, neurodegenerative disease [1, 2]. An outstanding pathological feature of MS is demyelination of axons in certain areas of the brain and spinal cord (the CNS). The demyelination is associated with reactive gliosis and infiltration of peripheral immune cells (neuroinflammatory response), axon damage, oligodendrocyte loss [3]. Most MS patients (approximately 85%) begin with the form of relapsing-remitting MS (RRMS), characterized by demyelination followed by periods of remission. At this stage, treatments based on managing neuroinflammation mediated by peripheral immune cells could facilitate neuronal repair, including remyelination of demyelinated axons, resulting in improvement and even disappearance of initial neurological symptoms [4].

Gradually, RRMS progresses into secondary-progressive MS, with limited remyelination and permanent neurological disability [5, 6]. Then treatments based on manipulating immune cell functions are no longer effective [7, 8], thus making managing neurological symptoms difficult. Studies suggest that promoting oligodendrocyte regeneration and thus remyelination could prevent axonal degeneration and loss, thus providing significant benefits to MS patients [9, 10]. Remyelination in the CNS depends on the proliferation and differentiation of oligodendrocyte precursor cells (OPCs) [7]. In MS lesions, the OPCs exist in the areas surrounding demyelinated lesions (albeit in reduced numbers in some cases), but they somehow fail to mature to oligodendrocytes capable of producing myelin [11, 12]. Developing therapeutic drugs that directly target OPC differentiation and oligodendrocyte myelination is hindered by the lack of animal models that recapitulate the core chronic MS pathologies, particularly with prolonged demyelination and remyelination in the context of chronic neuroinflammation [13].

The current MS-related animal models have made significant contributions to the understanding of MS pathogenesis and the development of therapeutic interventions, but they also have significant limitations. EAE (experimental autoimmune encephalomyelitis) model, based on immunization with myelin-oligodendrocyte glycoprotein (MOG) [14], is commonly used for the study of MS pathology from an inflammatory perspective, but the demyelination primarily occurs in the spinal cord and demyelination/remyelination in EAE is always unpredictable, which makes it difficult to study the process of demyelination/remyelination [15]. The cuprizone demyelination model has been frequently used to study demyelination and remyelination, but demyelination occurs less uniformly in the brain and demyelination is often compounded by the remyelination process despite the continued consumption of cuprizone and the remyelination process completes relatively fast [16].

Now we report the generation and characterization of a new demyelination model based on repeated ablations of mature Tmem10^+^ oligodendrocytes. This model not only presents the core pathological features of MS but manifests certain unique features, making it particularly useful to assess the efficacy of therapeutic interventions to promote remyelination under demyelinated conditions. We show that this model manifests a prolonged state of demyelination and axonal injury, which is accompanied by chronic microglial/inflammatory response with lymphocyte infiltration.

## Results

### Tmem10^+^ oligodendrocytes loss and subsequent repopulation following repeated DT treatments

Tmem10, also known as Opalin [17], is a transmembrane glycoprotein whose expression is confined to the relatively late stage of mature oligodendrocytes in the brain and spinal cord. Expression of Tmem10 lags the expressions of myelin-associated glycoprotein (MAG) and myelin basic protein (MBP) [18]. Widespread loss of Tmem10^+^ cells including demyelinated lesions and normal-appearing white matter has been identified in MS patients [19]. Previous studies have shown that disrupting the Tmem10 gene in mice had no obvious impacts on developmental myelination [20]. To ablate Tmem10^+^ mature oligodendrocytes, we crossed Rosa26iDTR mouse [21] with Tmem10-Cre mouse (Suppl. Fig. 1A, B), resulting in the double transgenic mouse in which the diphtheria toxin receptor (DTR) was selectively expressed in mature oligodendrocytes. The expression of DTR in Tmem10^+^ oligodendrocytes rendered them sensitive to diphtheria toxin (DT); therefore, the injection of DT would cause apoptotic death of Tmem10^+^ oligodendrocytes (Suppl. Fig. 1C).

The Tmem10^+^ oligodendrocyte-specific expression of DTR was indicated by selective expression in oligodendrocytes of tdTomato reporter in mice with Tmem10-Cre; Rosa26iDTR; Rosa-tdTomato. In this triple transgenic mouse, nearly 96% and 98% tdTomato^+^ cells were CC1^+^ differentiated oligodendrocytes in the corpus callosum and cortex, respectively (Suppl. Fig. 1D, E). Tmem10^+^ oligodendrocytes accounted for about 50% to 60% of the total pool of differentiated oligodendrocytes (CC1^+^) in the corpus callosum (CC) and cortex of the mouse brain (Suppl. Fig. 1F). Furthermore, we did not observe tdTomato immunolabeling of Iba1^+^ microglia or Sox9^+^ astrocytes (Suppl. Fig. 1G). Rare tdTomato^+^ cells were NeuN^+^ neurons in cortex (Suppl. Fig. 1G).

**Fig. 1:**
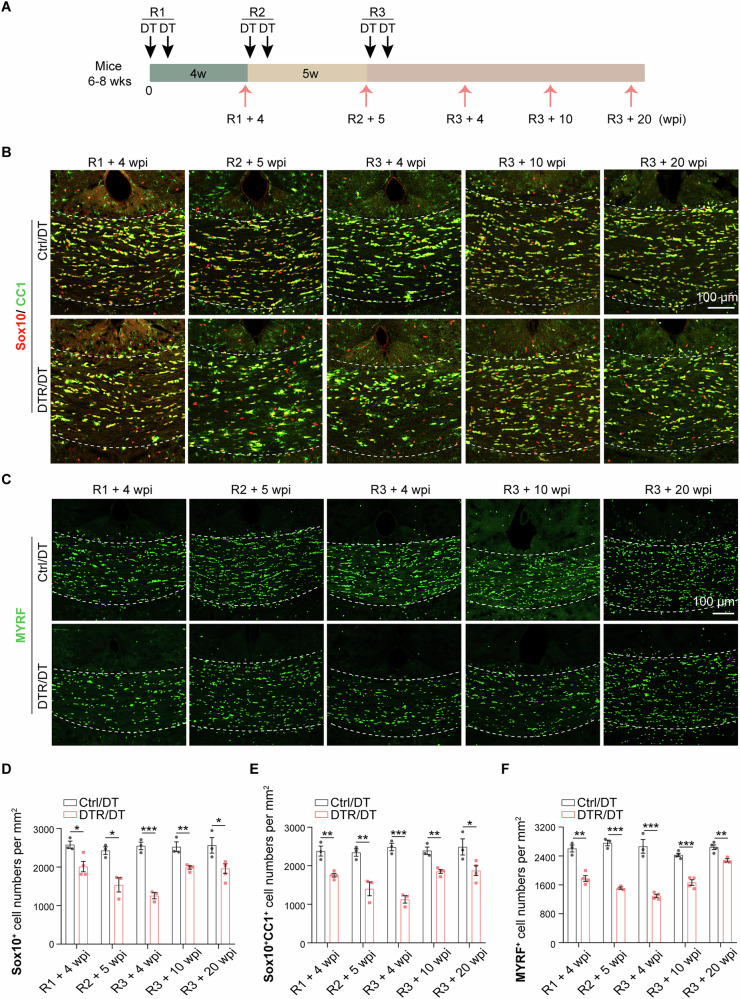
Oligodendrocytes loss and subsequent repopulation following repeated DT treatments. **A** Schematic diagram of three rounds of DT treatment (black arrows) and timepoints of animals sacrificed (pink arrows) to detect mature oligodendrocyte dynamics. R1: the 1^st^ round of DT injection. R2: the 2^nd^ round of DT injection. R3: the 3^rd^ round of DT injection. wpi: weeks post DT injection. **B**, **D**, **E** Immunostaining and quantification of Sox10/CC1 indicated the dynamic changes of Sox10^+^ and Sox10^+^CC1^+^ cells in the corpus callosum of DTR/DT mice following repeated DT injections. (*n* = 3–4 mice per group, scale bar = 100μm, mean ± SEM, *p < 0.05, **p < 0.01, ***p < 0.001, two-tailed *t* test). **C**, **F** Immunostaining and quantification of MYRF indicated the dynamic changes of MYRF^+^ differentiated oligodendrocytes in the corpus callosum of DTR/DT mice following repeated DT injections. (*n* = 3–4 mice per group, scale bar = 200μm, mean ± SEM, **p < 0.01, ***p < 0.001, two-tailed *t* test).

As the etiology of MS in most cases is characterized by repeated injuries to oligodendrocytes and myelin [22], we wondered if multiple rounds of ablatingTmem10^+^ oligodendrocyte in mouse would yield a demyelination model that capitulates the core MS pathologies and is better suited for studying the dynamics of oligodendrocyte regeneration and remyelination. Therefore, we carried out multiple rounds (up to 3 rounds) of DT injection to the 6-8-week Tmem10-cre; Rosa26iDTR mice (DTR mice), with each round consisting of 2 small doses (25 ng DT/kg), as illustrated in Fig. 1A. As control, age-matched Rosa26iDTR mice without Cre transgene (control mice) were also injected with DT in the same manner.

To validate the ablation of Tmem10^+^ oligodendrocytes, we assessed the expression of Sox10, a transcription factor that is expressed in the entire oligodendrocyte lineage [23] and CC1 marker for differentiated oligodendrocytes. The 1^st^ round of DT injection reduced Sox10^+^CC1^+^ oligodendrocytes to 74% in the corpus callosum (Fig. 1B, E) and 80% in the cortex (Suppl. Fig. 2A, D) of DTR/DT mice assessed 4 weeks after the initiation of the DT injection, respectively. At this time point, we conducted the 2^nd^ round of oligodendrocyte ablation by injecting DT, as we did with the 1^st^ round. By the 5^th^ week after the 2^nd^ round of ablation, the number of Sox10^+^CC1^+^ oligodendrocytes reduced to 60% in the corpus callosum (Fig. 1B, E) and 55% in the cortex (Suppl. Fig. 2A, D) of DTR/DT mice. At this point, we started the 3^rd^ round of DT injection. The density of Sox10^+^CC1^+^ oligodendrocytes reduced to 45% in the corpus callosum by 4 weeks after the 3^rd^ round of DT injection, then began to increase and restored to 77% of normal control level by 10 weeks after the 3^rd^ round of ablation (Fig. 1B, E). The production of Sox10^+^CC1^+^ differentiated oligodendrocytes appeared to stagnate over the next 10 weeks, as the number of Sox10^+^CC1^+^ cells was not changed until 20 weeks after the 3^rd^ round of ablation (Fig. 1B, E). In parallel, we examined the dynamic changes of differentiated oligodendrocytes in the cortex of DTR mice following the 3^rd^ round of DT injection. The number of Sox10^+^CC1^+^ oligodendrocytes decreased to 36% of normal control level 4 weeks after the 3^rd^ round of DT injection (Suppl. Fig. 2A, D), then the repopulation of differentiated oligodendrocytes increased gradually from 4 weeks to 20 weeks: by 10 weeks, the number of Sox10^+^CC1^+^ cells was 51% of normal control level, and further increased to 65% of normal control level by 20 weeks after the 3^rd^ round of DT injection (Suppl. Fig. 2A, D). The dynamics of Sox10^+^ cells were in accordance with Sox10^+^CC1^+^ oligodendrocytes both in the cortex and corpus callosum of DTR/DT mice following repeated DT injections (Fig. 1D, Suppl. Fig. 2C).

To further examine the dynamic changes of oligodendrocytes in the demyelination model, we performed immunostaining to assess the mature myelin-forming oligodendrocytes that express the myelin regulatory factor gene (MYRF), an essential transcriptional factor which is required for oligodendrocyte differentiation and CNS myelination [24, 25]. In the corpus callosum of DTR/DT mice, the number of MYRF^+^ cells decreased to 68% of control/DT mice 4 weeks after one round of DT injection (Fig. 1C, F). In a separate set of experiments where mice were subjected to two rounds of DT injection, MYRF^+^ oligodendrocytes were reduced to 54% of the normal control level 5 weeks after the 2^nd^ round of DT injection (Fig. 1C, F).

Following the third round of DT injection, the number of MYRF^+^ cells was 48% of control/DT mice 4 weeks later, and then recovered to 68% of control level by 10 weeks after the 3^rd^ round of DT injection (Fig. 1C, F). The number of MYRF^+^ cells reached 86% of normal level at 20 weeks after the 3^rd^ round of DT injection (Fig. 1C, F). In the cortex of DTR/DT mice, the changes to MYRF^+^ cells manifest the same pattern as those in the corpus callosum (Suppl. Fig. 2B, E). In general, our results showed that the dynamic changes of MYRF^+^ cells both in the cortex and in the corpus callosum of DTR/DT mice are consistent with those of SOX10^+^CC1^+^ cells following repeated DT injections.

### Persistent neuroinflammation including gliosis and lymphocyte infiltration following oligodendrocyte ablations

**Fig. 2:**
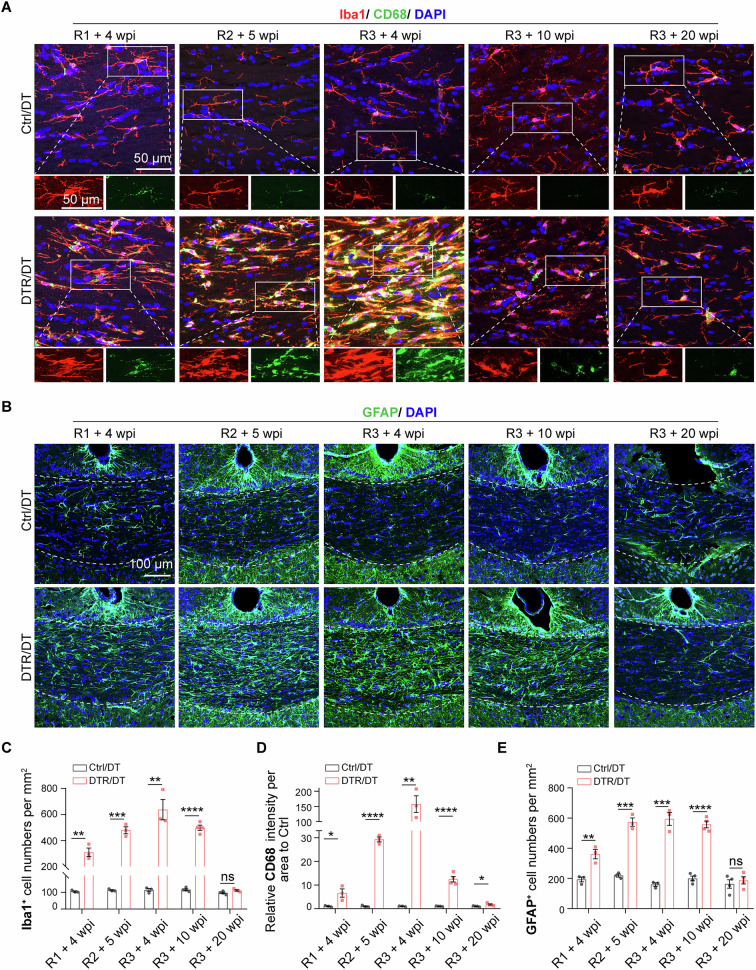
Chronic neuroinflammatory responses following repeated oligodendrocyte ablations. **A**, **C**, **D** Confocal images and quantification of Iba1/CD68 staining at different timepoints demonstrated that microglia sustained reactive state in the corpus callosum once sensitized by oligodendrocyte death, suggested by increased numbers of Iba1^+^ microglia (C) and CD68 intensities(D). Then at 20 weeks after the 3^rd^ round of DT injection, the numbers of Iba1^+^ microglia in DTR/DT mice were comparable to normal control level, however, the intensity of CD68 in the corpus callosum of DTR/DT mice was still higher than that in control/DT mice. (*n* = 3–4 mice per group, scale bar = 50μm, mean ± SEM, *p < 0.05, **p < 0.01, ***p < 0.001, ****p < 0.0001, ns, not significant, two-tailed *t* test). White box showed an enlarged graph of Iba1^+^/CD68^+^ microglia. **B**, **E** Confocal images and quantification of GFAP staining in the corpus callosum at different timepoints suggested that astrocytes of DTR/DT mice sustained reactive state once activated by oligodendrocyte death, finally returned to normal control level at 20 weeks after the 3^rd^ round of DT injection (*n* = 3–4 mice per group, scale bar = 100μm, mean ± SEM, **p < 0.01, ***p < 0.001, ****p < 0.0001, ns, not significant, two-tailed *t* test).

To examine if persistent inflammation characterized by robust reactive gliosis and lymphocyte infiltration occurs following repeated oligodendrocyte ablations, we first performed immunostaining to quantify reactive microglia and astrocytes in the cortex and corpus callosum. To identify reactive microglia, we stained brain tissues with anti-Iba1 antibody that detects a calcium-binding protein in microglia and a small percentage of macrophages in the brain and increased Iba1 expression is indicative of microglial reactivity [26]. In addition, we stained CD68, a lysosomal membrane protein whose increase is often correlated with the enhanced activity of reactive microglia [27, 28], which could contribute to tissue damage, altering disease outcome. We found that after the 1^st^ round of DT injection into the DTR mouse, the number of Iba1^+^ microglia significantly increased in the corpus callosum and cortex (Fig. 2A, C, Suppl. Fig. 3A, C). In those Iba1^+^ microglia, CD68 was also increased (Fig. 2A, D, Suppl. Fig. 3A, D). In parallel, we immunostained with an antibody against the astrocyte marker GFAP (glial fibrillary acidic protein). While GFAP is readily detectable in astrocytes in the white matter, e.g., corpus callosum, it is not normally detectable in astrocytes present in the gray matter, e.g., cortex. When astrocytes in the cortex and other regions become reactive in response to tissue injury, they manifest robust GFAP reactivity [29]. We identified strong GFAP reactivity both in the corpus callosum and cortex after the 1^st^ round of DT injection/oligodendrocyte ablation (Fig. 2B, E, Suppl. Fig. 3B, E). Thus, reactive gliosis was strong in the DTR/DT mouse.

The reactive gliosis in the DTR/DT mice was sustained by subsequent rounds of oligodendrocyte ablations (Fig. 2, Suppl. Fig. 3). By 10 weeks after the 3^rd^ round of oligodendrocyte ablation, we still detected robust reactive microglia and astrocytes (Fig. 2, Suppl. Fig. 3). By 20 weeks after the 3^rd^ round of oligodendrocyte ablation, the number of microglia and reactive astrocyte in the corpus callosum and cortex returned to normal control level (Fig. 2, Suppl. Fig. 3), the intensity of CD68 was still significantly higher than that of age-matched control in the corpus callosum (Fig. 2A, D), suggesting persistent microglia reactivity in the oligodendrocyte ablation/demyelination model.

**Fig. 3:**
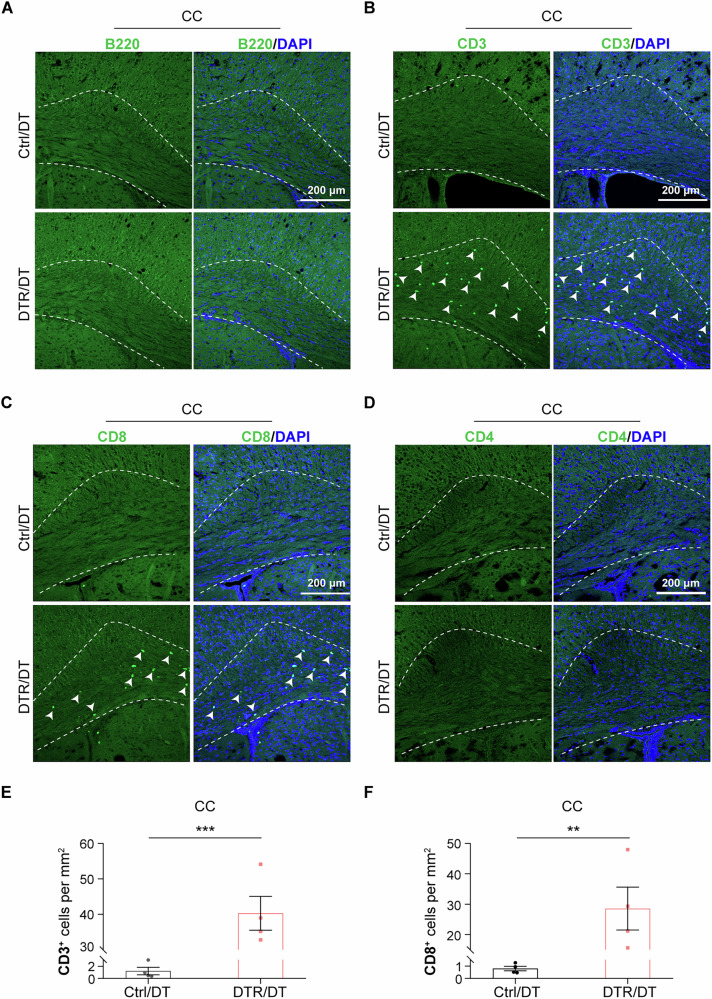
Repeated oligodendrocyte ablations triggered T immune cells infiltration into white matter of the DTR mice. **A** B220 (a specific marker of B cells) immunostaining revealed no apparent B immune cells infiltrated into the corpus callosum of DTR/DT mice at 4 weeks after the 3^rd^ round of DT injection. (*n* = 4 mice per group, scale bar = 200μm). **B**, **E** Confocal images of CD3 (a specific marker of T cells) immunostaining and quantification suggested that CD3^+^ T immune cells infiltrated into corpus callosum of DTR/DT mice at 4 weeks after the 3^rd^ round of DT injection. (*n* = 4 mice per group, scale bar = 200μm, mean ± SEM, ***p < 0.001, two-tailed *t* test). **C**, **F** Representative images of CD8 immunostaining and quantification showed that it was mainly CD8^+^ lymphocytes that pervaded into the corpus callosum of DTR/DT mice at 4 weeks after the 3^rd^ round of DT injection. (*n* = 4 mice per group, scale bar = 200 μm, mean ± SEM, **p < 0.01, two-tailed *t* test). **D** Representative images of CD4 immunofluorescence showed few CD4^+^ cells in the corpus callosum of DTR/DT mice at 4 weeks after the 3^rd^ round of DT injection. (*n* = 4 mice per group, scale bar = 200μm).

Lymphocyte infiltration is also an important component of inflammatory response present in MS and MS-related animal models, e.g., EAE model [30, 31]. To examine if lymphocyte infiltration was also present in the demyelination model, we performed immunostaining with antibodies against T and B lymphocyte markers in the brain sections of DTR/DT and control/DT mice. Albeit without apparent presence of B220^+^ B lymphocytes (Fig. 3A), CD3^+^ T lymphocytes were detected in the white matter of the DTR/DT mice subjected to 3 rounds of oligodendrocyte ablation (Fig. 3B, E). In the DTR/DT brain, infiltrating T lymphocytes were found mainly in the white matter, but not in the gray matter. Most of these T lymphocytes were CD8^+^ lymphocytes (Fig. 3C, F), except for a few CD4^+^ T helper lymphocytes (Fig. 3D), which is consistent with the clinical finding that CD8^+^ immune cells are the dominant subtype of T lymphocytes that infiltrate into the CNS lesions in MS patients [32, 33]. By 20 weeks after the 3^rd^ round of DT injection, we found few T cells in the white matter of DTR/DT mice (Suppl. Fig. 4), suggesting infiltrated T lymphocytes gradually disappeared at the remyelination phase.

### Prolonged demyelination followed by spontaneous remyelination after repeated Tmem10^+^ oligodendrocyte ablations

To determine if demyelination occurs after each round of oligodendrocyte ablation, we performed Western blotting to assess the changes to multiple myelin proteins, and Black Gold staining and LFB staining to examine the extent of myelination 4-5 weeks after the completion of each round of oligodendrocyte ablation. We chose this time point because demyelination was noted in various acute demyelination models [34, 35]. Unexpectedly, we did not find apparent demyelination in the brain of the DTR/DT mouse at 4 weeks and even a later timepoint after the 1^st^ round of DT injection, as indicated by comparable amounts of myelin related proteins between control/DT and DTR/DT mice, as well as Black Gold staining and LFB staining (Suppl. Fig. 5). To further confirm that one round of DT injection/oligodendrocyte ablation is not sufficient to cause significant demyelination under our experimental condition, we injected DT to 15-17-week-old DTR-expressing mice in the same manner (Suppl. Fig. 6A). Still, no apparent demyelination was detected at this timepoint (Suppl. Fig. 6B–F).

Then we asked if two rounds of oligodendrocyte ablations would cause demyelination (Suppl. Fig. 7A). Five weeks after the 2^nd^ of DT injection, no significant demyelination was detected in the DTR/DT mice, by Western blotting of myelin proteins of the cortex (Suppl. Fig. 7B, C), Black Gold staining and LFB staining (Suppl. Fig. 7F, G). However, we found reduced myelin proteins and modestly reduced staining intensities in Black Gold staining at 9 weeks after the 2^nd^ round of DT injections, suggesting that modest demyelination occurred in the DTR/DT mice at this time point (Suppl. Fig. 7D, E, H, I).

**Fig. 4:**
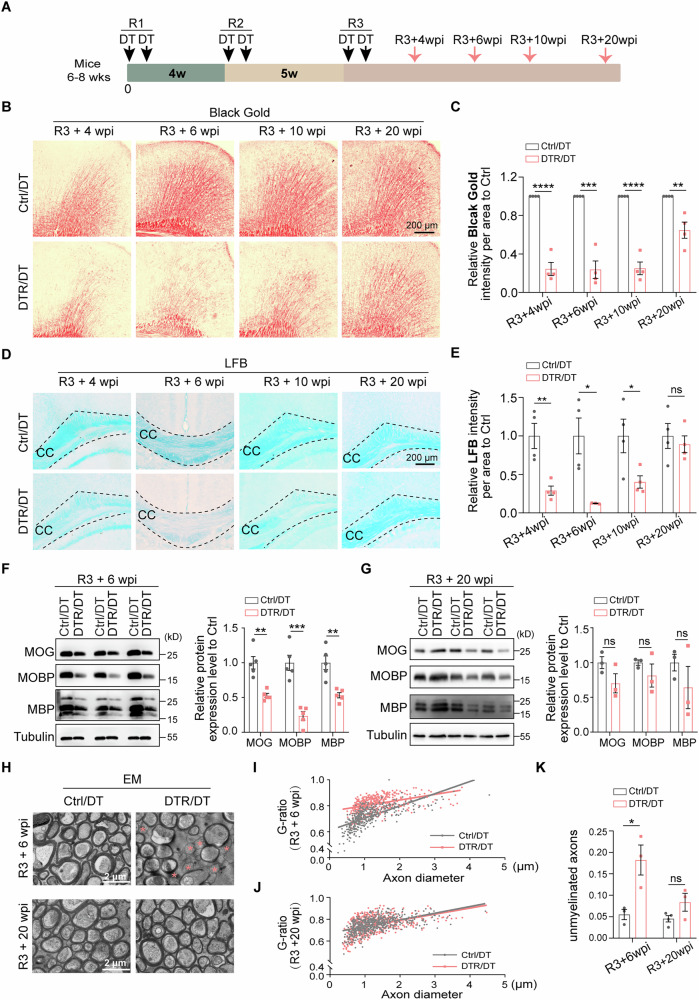
Three rounds of oligodendrocyte ablation resulted in significant and prolonged demyelination followed by spontaneous remyelination. **A** Schematic diagram of timepoints (pink arrows) to examine myelin content. **B**, **C** Representative images and quantification of Black Gold staining at various timepoints after the 3^rd^ round of DT injection showed the myelin content reduced to 24% in the cortex of DTR/DT mice at 4 weeks after the 3^rd^ round of oligodendrocyte ablation, the intensity of Black Gold staining remained the same low level by 10 weeks after the 3^rd^ round of DT injection, then the intensity of Black Gold staining was restored to 64% of normal control level. (*n* = 3-4 mice per group, scale bar = 200 μm, mean ± SEM, **p < 0.01, ***p < 0.001, ****p < 0.0001, two-tailed *t* test). **D**, **E** Representative images and quantification of LFB staining at various timepoints after the 3^rd^ round of DT injection showed the myelin content reduced to 28% in the corpus callosum of DTR/DT mice at 4 weeks after the 3^rd^ round of oligodendrocyte ablation, in the following 6 weeks, the intensity of LFB staining kept a comparably low level, then the intensity LFB staining totally was restored to normal control level at 20 weeks after the 3^rd^ round of DT treatment. (*n* = 3–4 mice per group, scale bar = 200μm, mean ± SEM, *p < 0.05, **p < 0.01, ns, not significant, two-tailed *t* test). **F, G**. Western blotting and quantification showed a dramatic reduction of myelin related proteins including MOG, MOBP, MBP in the cortex of DTR/DT mice compared with control/DT mice at 6 weeks after the 3^rd^ round of DT injection, the expression of myelin related proteins MOG, MOBP and MBP restored to 70%, 81% and 64% of normal control level in the cortex of DTR/DT mice at 20 weeks after 3^rd^ round of DT administration (*n* = 3–5 mice per group, mean ± SEM. **p < 0.01, ***p < 0.001, ns, not significant, two-tailed *t* test). **H–K** Representative EM images (**H**) and G-ratio quantification of optic nerves and indicated myelinated axons became thinner (**I**) and the naked axons increased (**K**) in DTR/DT mice compared with control/DT mice at 6 weeks after the 3^rd^ round of oligodendrocyte ablation. The myelin thickness (**H**, **J**) and ratios of unmyelinated axons (**K**) of optic nerve between control/DT and DTR/DT mice had no obvious difference at 20 weeks after the 3^rd^ round of DT injection. (*n* = 3-4 mice per group, scale bar = 2.0 μm, mean ± SEM, *p < 0.05, ns, not significant, two-tailed *t* test). ON: optic nerve. EM electron microscopy.

The 3^rd^ round of oligodendrocyte ablation with DT injection produced more robust demyelination (Fig. 4). As indicated by decreased Black Gold and LFB staining, reduced myelin content was noted in the cortex and corpus callosum by 4 weeks after the 3^rd^ round of oligodendrocyte ablation (Fig. 4B–E). By 10 weeks after the 3^rd^ round of DT injection, the intensity of Black Gold and LFB staining remained at a comparably low level, then the intensity of Black Gold and LFB staining was restored to 64% and 90% of the normal control level by 20 weeks after the 3^rd^ round of DT injection, respectively (Fig. 4B-E). Consistent with reduced Black Gold and LFB staining, we found that multiple myelin proteins such as MOG (myelin oligodendrocyte glycoprotein), MOBP (Myelin-associated Oligodendrocyte Basic Protein) and MBP (Myelin Basic Protein) were significantly reduced to 52%, 23% and 53% of normal control level by 6 weeks after the 3^rd^ round of ablation (Fig. 4F, G). By 20 weeks after the 3^rd^ round of ablation, myelin proteins such as MOG, MOBP and MBP were restored to 70%, 81% and 64% of normal control level, respectively (Fig. 4F, G). To further validate the demyelination/remyelination caused by multiple rounds of oligodendrocyte ablation, we performed electron microscopy (EM) analysis of optic nerves of DTR/DT mice at various time points (Fig. 4H–K). By 6 weeks after the 3^rd^ round of ablation, the demyelination in the optic nerves was striking: while almost all axons of the optic nerves from control/DT mice were myelinated at this age, ~18% of axons from the DTR/DT mice were unmyelinated (Fig. 4K). Those myelinated axons had much thinner myelin wraps, as indicated by increased “g” ratios (the ratio of the inner diameter to the outer diameter of the axon) (Fig. 4H, I). In addition, EM analysis of corpus callosum of DTR/DT mice showed that myelinated axons were significantly reduced and the G-ratio of axons with myelin membranes were increased significantly, which is consistent with the results obtained by EM of optic nerves (Suppl. Fig. 8). However, by 20 weeks after the 3^rd^ round of ablation, the percentage of myelinated axons were returned to normal control level (Fig. 4K), demyelinated axons were remyelinated as the myelin “g” ratios in DTR/DT mice were comparable to control/DT mice (Fig. 4H, J). All these results suggest that there exists a long “time window” between the onset of demyelination and the almost completion of spontaneous remyelination in this new demyelination model.

### Axonal damage following repeated oligodendrocyte ablations

**Fig. 5:**
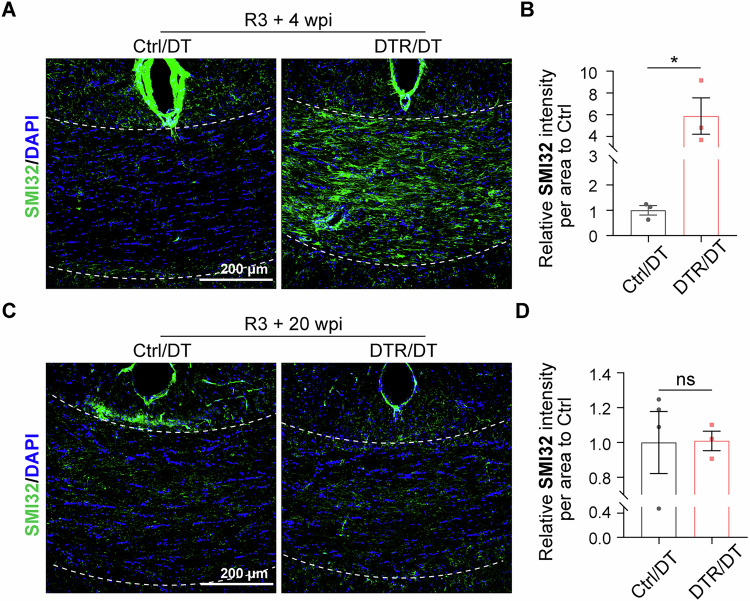
Repeated oligodendrocyte ablations resulted in axonal injury. **A**, **B** SMI32 immunostaining and quantification suggested the intensity of SMI32 in the corpus callosum of DTR/DT mice was significantly increased compared with control/DT mice at 4 weeks after the 3^rd^ round of DT injection. (*n* = 3 mice per group, scale bar = 200 μm, mean ± SEM, *p < 0.05, two-tailed *t* test). **C**, **D** SMI32 immunostaining and quantification indicated the intensity of SMI32 in the corpus callosum of DTR/DT mice was comparable to normal control level at 20 weeks after the 3^rd^ round of DT injection. (*n* = 3-4 mice per group, scale bar = 200μm, mean ± SEM, ns not significant, two-tailed t test).

Axon demyelination and neuroinflammation are fundamental errors behind axon injury and loss in MS lesions [36–38], which are tightly correlated with disease progression and irreversible neurological disability in MS [39]. To assess axon damage in the demyelination model, we examined the immunoreactivity of axon SMI32 in the corpus callosum. SMI32 antibody normally detects non-phosphorylated neurofilaments that are abundant in neuronal soma and dendrites. In normally myelinated axons, neurofilaments are heavily phosphorylated with little SMI32 immunoreactivity. When axon damage occurs, SMI32 reactivity could be significantly increased. Indeed, increased SMI32 is noted in axons within MS lesions [40]. In the normal control/DT mice, we found little SMI32 reactivity in the corpus callosum, but in the DTR/DT mice, a significant increase in SMI32 was present in the corpus callosum 4 weeks after the 3^rd^ round of DT injection (Fig. 5A, B). This finding supports the notion that axon damage occurs in the demyelination model. 20 weeks after the 3^rd^ round of DT injection, SMI32 reactivity was reduced to the normal control level (Fig. 5C, D), suggesting axon recovery from the damage caused presumably by demyelination and inflammatory response. Of note, the axon recovery was correlated with significant recovery of demyelination in this region and reduced inflammatory response as indicated by reactivity of microglia and astrocytes.

### Intact OPCs response following repeated oligodendrocyte ablations

**Fig. 6:**
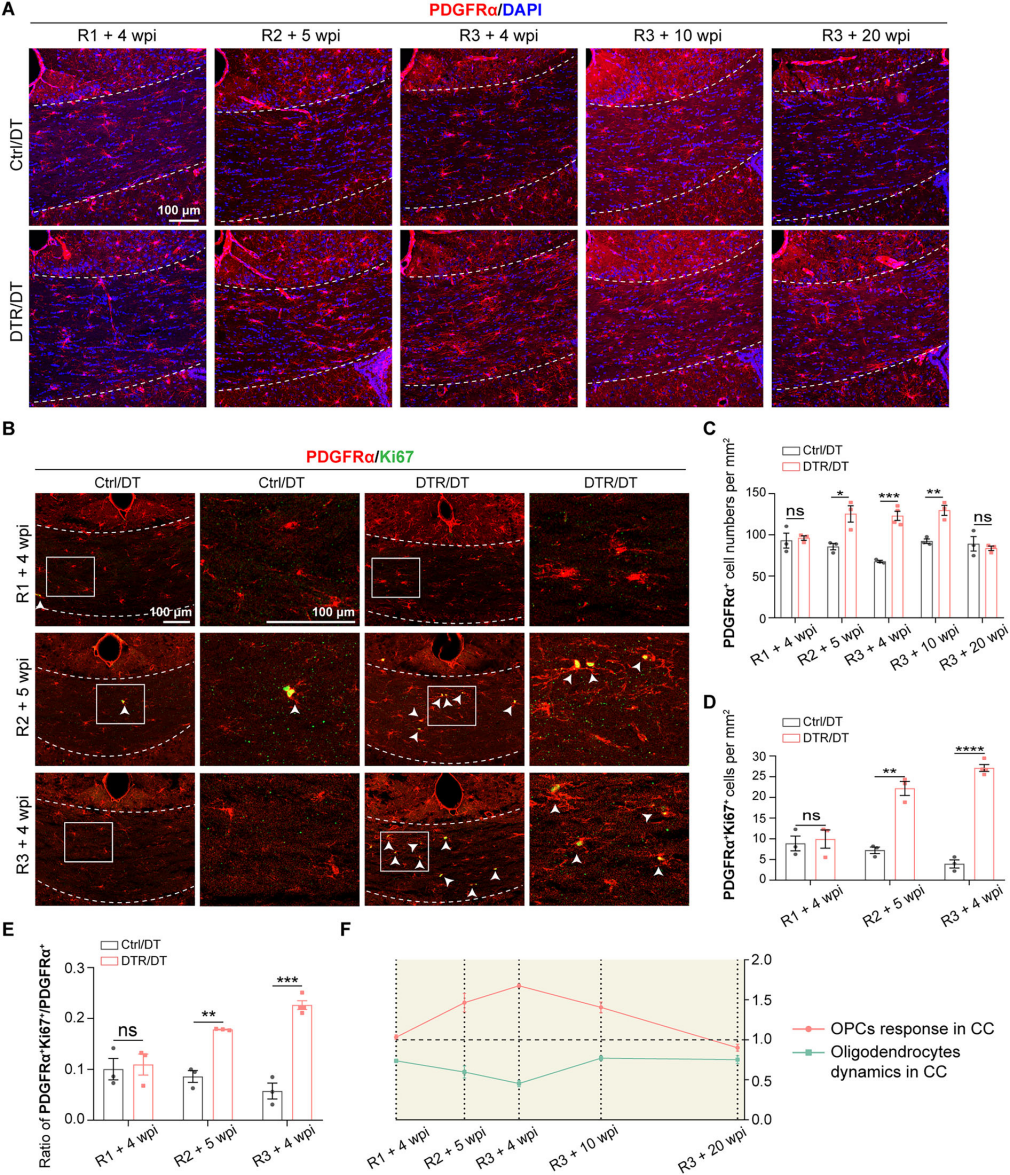
Enhanced OPCs proliferation in the corpus callosum of DTR/DT mice in response to repeated Tmem10^+^ oligodendrocyte ablations. **A**, **C** Representative images and quantification of PDGFRα immunostaining showed the number of PDGFRα^+^ OPCs in the corpus callosum of DTR/DT mice was comparable to that of control/DT mice 4 weeks after the 1^st^ round of DT injection, and then increased significantly by 5 weeks after the 2^nd^ round of DT injection. Following the 3^rd^ round of DT injection, the number of OPCs of DTR/DT mice was continuously greater than that of control/DT mice 4 weeks later, and then gradually declined and returned to the basal level by 20 weeks after the 3^rd^ round of DT injection. (*n* = 3-4 mice per group, scale bar = 100 μm, mean ± SEM, *p < 0.05, **p < 0.01, ***p < 0.001, ns, not significant, two-tailed *t* test). **B**, **D** Immunostaining of PDGFRα/Ki67 and quantification showed the number of double-positive cells expressing PDGFRα and Ki67 in the corpus callosum of DTR/DT mice remained unchanged at 4 weeks after the 1st round of DT injection, then increased sharply 5 weeks later following the 2nd round of DT injection. By 4 weeks after the 3^rd^ round of DT injection, the number of PDGFRα^+^Ki67^+^ cells in the corpus callosum of DTR/DT mice far exceeded that of control/DT mice. (*n* = 3–4 mice per group, scale bar = 100 μm, mean ± SEM, **p < 0.01, ****p < 0.00001, ns, not significant, two-tailed *t* test). **E** Quantification of the ratio of PDGFRα^+^Ki67^+^/PDGFRα^+^ in the corpus callosum of DTR/DT mice and control/DT mice following repeated oligodendrocyte ablations. (*n* = 3–4 mice per group, scale bar = 100 μm, mean ± SEM, **p < 0.01, ***p < 0.001, ns not significant, two-tailed *t* test). **F** The short thumbnail shows that following repeated oligodendrocyte ablations, the dynamic change of OPC responses in the corpus callosum of DTR/DT mice presented a contrary trend to that of differentiated oligodendrocytes.

Upon oligodendrocyte injury and demyelination, OPCs are recruited by proliferation and migration into injury areas and then differentiate into mature, myelinating oligodendrocyte for remyelination [41]. To examine how adult OPCs respond to repeated ablations of oligodendrocyte, we assessed the number of OPCs in DTR/DT mice at various time points after each round of oligodendrocyte ablation. In the corpus callosum, we did not detect obvious difference in the number of OPCs (indicated by PDGFRα^+^) of DTR/DT mice compared with control/DT mice at 4 weeks after the 1^st^ round of oligodendrocyte ablation (Fig. 6A, C). The density of adult OPCs of DTR/DT mice was significantly higher than that of control/DT mice by 5 weeks after the 2^nd^ round of oligodendrocyte ablation (Fig. 6A, C). Following the 3^rd^ round of DT injection, the numbers of OPCs of DTR/DT mice were continuously more than that of control/DT mice 4 weeks later and then subsequently declined to normal control level at 20 weeks after the 3^rd^ round of DT injection (Fig. 6A, C). In parallel, we examined the OPCs responses in the cortex of DTR/DT mice following repeated oligodendrocyte ablations. We only detected a slight increase in the number of OPCs by 5 weeks after the 2^nd^ round of oligodendrocyte ablation, and the increase was maintained for a relatively short period of time. By 4 weeks after the 3^rd^ round of oligodendrocyte ablation, the number of OPCs was back down to the normal control level (Suppl. Fig. 9A, C).

Next, to examine OPC proliferation, we performed Ki67 staining that labels cells in the active cell cycle. Consistently, we found that the PDGFRα^+^Ki67^+^ cell population in the corpus callosum of DTR/DT mice was not affected at 4 weeks after the 1^st^ round of DT injection, but then far exceeded that of control/DT mice at 5 weeks after the 2^nd^ round of DT injection (Fig. 6B, D). By 4 weeks after the 3^rd^ round of DT injection, the number of PDGFRα^+^Ki67^+^ OPCs in the corpus callosum of DTR/DT mice was consistently higher than that of control/DT mice (Fig. 6B, D). To further assess the proliferation rate of OPCs, we analyzed the ratio of PDGFRα^+^Ki67^+^/PDGFRα^+^ at different time points following repeated oligodendrocyte ablations. In the corpus callosum of DTR/DT mice, we found that the proliferation rate of OPCs was not altered at 4 weeks after the 1^st^ round of oligodendrocyte ablation (Fig. 6E). However, the proliferation rate of OPCs indicated by the ratio of PDGFRα^+^Ki67^+^/PDGFRα^+^, was significantly accelerated at 5 weeks after the 2^nd^ round of oligodendrocyte ablations (Fig. 6E). The OPC proliferation rate in the corpus callosum of DTR/DT mice was still higher than that in control/DT mice at 4 weeks after the 3^rd^ round of oligodendrocyte ablations (Fig. 6E). In the cortex, there was no difference in the number of PDGFRα^+^Ki67^+^ OPCs (Suppl. Fig. 9B, D) and the ratio of PDGFRα^+^ Ki67^+^/ PDGFRα^+^ (Suppl. Fig. 9B, E) between DTR/DT and control/DT mice following repeated oligodendrocyte ablations. These results suggested a regional difference in OPCs proliferation rate: the proliferation rate of OPCs in the corpus callosum was higher than that in the cortex.

To examine the differentiation of OPCs and newborn differentiated oligodendrocytes in the demyelinated brain, we examined the marker BCAS1 (breast carcinoma amplified sequence 1) that has previously been reported to be selectively expressed in newly formed immature oligodendrocytes [42], a stage that separates OPCs from differentiated oligodendrocytes. Immunostaining showed that the numbers of Bcas1^+^ cells were increased in both the cortex and corpus callosum (compared with Ctrl/DT mice) of DTR/DT mice at 4 weeks and 10 weeks after the 3^rd^ round of DT injection. At 20 weeks after the 3^rd^ round of DT injection, the number of Bcas1^+^ cells in the cortex and corpus callosum of DTR/DT mice was comparable to that in Ctrl/DT mice (Suppl. Fig. 10).

The OPCs responses were correlated with the dynamic changes of mature oligodendrocytes (Fig. 6F, Suppl. Fig. 9F), as stated in Fig. 1, suggesting that the repopulation of oligodendrocytes following three rounds of oligodendrocyte ablation was a protracted process, and the repopulation of oligodendrocytes was more sluggish in the cortex than corpus callosum, suggesting region heterogeneity in OPC proliferation and differentiation.

### Use of the DTR/DT demyelination model for evaluating the efficacy of compounds towards remyelination

**Fig. 7:**
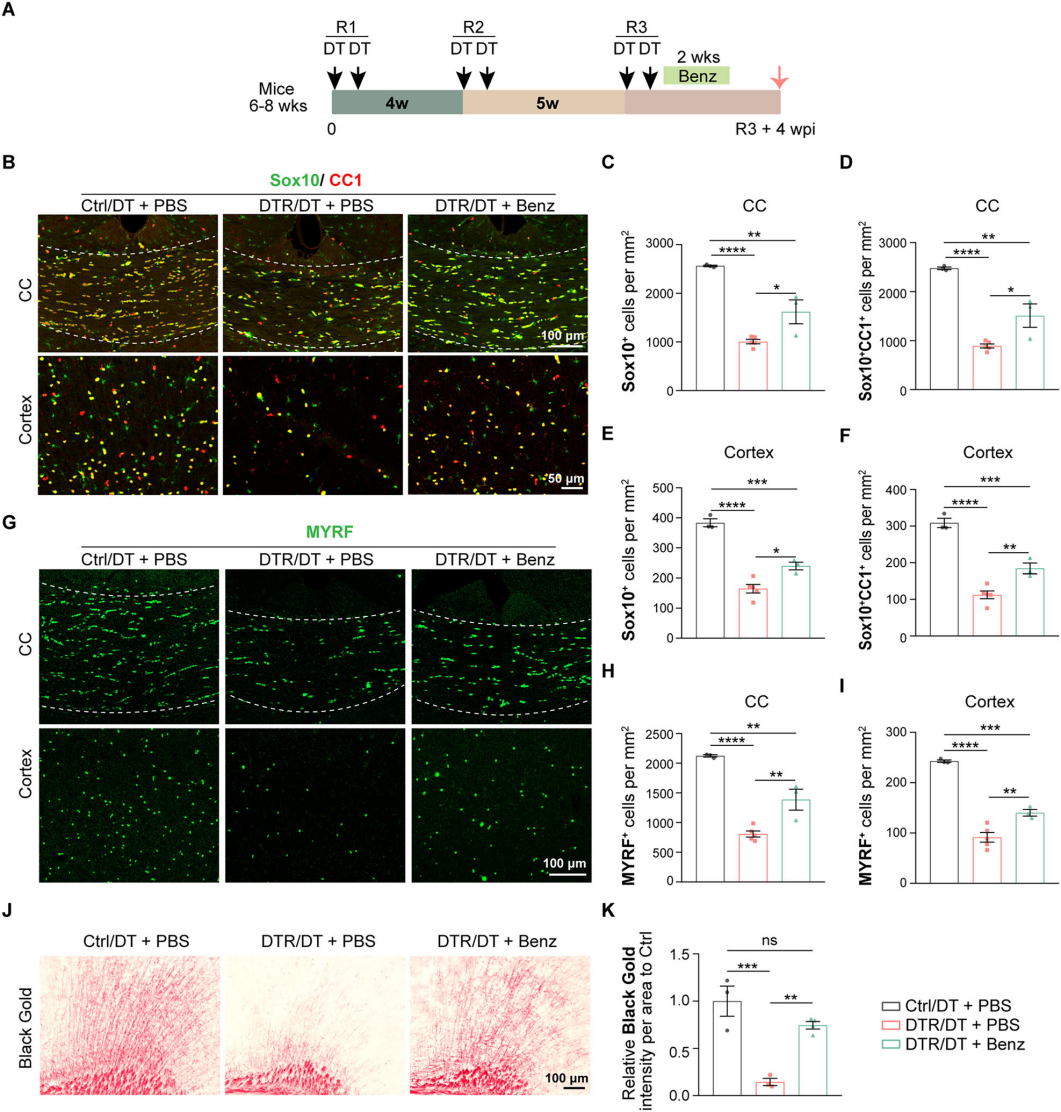
Benztropine could facilitate remyelination in the prolonged demyelination model. **A** Approaches of Benztropine (Benz) or PBS treatment and timepoints to analyze. **B–F** Immunostaining and quantification of Sox10/CC1 revealed a decrease in the density of Sox10^+^ and Sox10^+^CC1^+^ cells both in the corpus callosum and cortex of PBS-treated DTR/DT mice (DTR/DT + PBS group), compared with PBS-treated control/DT mice (control/DT + PBS group). The number of Sox10^+^ and Sox10^+^CC1^+^ cells oligodendrocytes increased significantly both in the corpus callosum and cortex of Benz-treated DTR/DT mice (DTR/DT + Benz group) compare with those treated with PBS (DTR/DT + PBS group). (*n* = 3–5 mice per group, scale bar = 100 μm(CC)/50μm (Cortex), mean ± SEM, *p < 0.05, **p < 0.01, ***p < 0.001, ****p < 0.0001, one-way ANOVA). **G–I** Immunostaining and quantification of MYRF demonstrated an increase in the density of MYRF^+^ differentiated oligodendrocytes in the cortex of Benztropine-treated DTR/DT mice (DTR/DT + Benz group) compared with those treated with PBS (DTR/DT + PBS group). (*n* = 3–5 mice per group, scale bar = 100 μm, mean ± SEM, **p < 0.01, ***p < 0.001, ****p < 0.0001, one-way ANOVA). **J**, **K** Black Gold staining demonstrated a dramatic increase of Black Gold intensity in Benztropine-treated DTR/DT mice (DTR/DT + Benz group), compared with PBS-treated DTR/DT mice (DTR/DT + PBS group), *n* = 3–4 mice per group, scale bar = 100 μm, **p < 0.01, ***p < 0.001, ns not significant, one-way ANOVA).

The prolonged process of regeneration of mature oligodendrocytes/myelin following the 3^rd^ round of oligodendrocyte ablation suggest it could provide an excellent “time-window” to evaluate the efficacy of potential therapeutic agents in promoting remyelination in demyelinating disorders, e.g., MS. To examine this notion, we assessed the effect of Benztropine on remyelination in this DTR/DT model. Benztropine has been reported to induce the proliferation and differentiation of OPCs through inhibiting M1/3 muscarinic receptor signaling pathway and had some efficacy of enhancing remyelination under certain conditions [43–46]. We started treating DTR/DT mice with Benztropine, right after the completion of the 3^rd^ round of DT injection (Fig. 7A). As control, a group of DTR/DT mice was treated with PBS that was used to dissolve Benztropine. The drug regiment lasted for 2 weeks with daily injection. Two weeks after the end of the drug regiment, we found that Benztropine treatment induced an increase in the number of Sox10^+^CC1^+^ oligodendrocytes both in the corpus callosum and cortex of DTR/DT mice (Fig. 7B–F). In addition, the amount of MYRF^+^ cells also increased significantly in the Benztropine-treated DTR/DT mice compared with those treated with PBS (Fig. 7G–I). Black Gold staining showed that Benztropine consistently enhanced myelination compared with PBS-treated DTR/DT mice (Fig. 7J, K).

Although the timing to start the drug treatment does not exclude the possibility that the treatment may help lessen demyelination, these results validate the effect of Benztropine to promote oligodendrocyte differentiation, enhancing myelin regeneration in vivo and support the idea that this novel prolonged demyelination model can be used as a tool to validate the efficacy of potential drugs targeting oligodendrocyte regeneration and remyelination.

## Discussion

Here we have generated a new demyelination/remyelination model with chronic neuroinflammation. The demyelination is caused by repeated (a total of 3 rounds in the space of about 9 weeks) genetic/pharmacological ablations of Tmem10^+^ oligodendrocytes. Unlike other reported oligodendrocyte ablation models [34, 35, 47, 48], this demyelination model survives long after the prolonged demyelination occurs and has a relatively long “time window” before spontaneous remyelination. This is in sharp contrast to most of other ablation models based on ablations of oligodendrocytes expressing MBP or MOG. [47, 48] These MBP- and MOG-based oligodendrocyte ablation models experience early life death due to severe neurological deficits, with most deaths occurring between 2 to 6 weeks after the initiation of the ablation. The exception is the oligodendrocyte ablation model based on the expression of the diphtheria toxin gene (DTA) under the control of PLP-CreER [34]. Some male PLP-CreER; DTA mice were noted to survive longer after oligodendrocyte ablation: all females died at 3 weeks after the initiation of ablation, but 70% of males survived longer than 3 weeks and recovered from most clinical symptoms by 10 weeks. However, these survivors developed fatal secondary neurological symptoms, and 50% of them would die at 52 weeks [49]. In contrast, nearly 90% DTR/DT mice, females and males, lived beyond 29 weeks after the initiation of the first round of oligodendrocyte ablation. Meanwhile, following three rounds of DT injection, all male and female DTR mice developed stable and persistent demyelination in consistent lesions, which would be most suitable for the study of the process of demyelination and remyelination.

Chronic neuroinflammation not only could play a role in the demyelination [50, 51], but also could significantly alter the regeneration of mature, myelin-forming oligodendrocytes, with implications to remyelination [52–54]. An outstanding feature of this Tmem10-based DTR/DT demyelination model is that the demyelination occurs in the context of chronic neuroinflammation with reactive gliosis (microglia and astrocytes) and lymphocyte infiltration. Reactive gliosis occurred upon the 1^st^ round of oligodendrocyte ablation and was maintained by subsequent rounds of oligodendrocyte ablations. Reaching its peak 4 weeks after the 3^rd^ round of oligodendrocyte ablations, the reactive state of microglia and astrocytes remained for a prolonged period: by 20 weeks after the 3^rd^ round of oligodendrocyte ablations, although the number of Iba1^+^ microglia and GFAP staining intensity were back to the normal control level, CD68 intensity was still higher than that of normal control microglia. Lymphocyte infiltration is another important factor in MS pathogenesis. Whether oligodendrocyte death by experimental ablation is sufficient to trigger lymphocyte infiltration in the brain remains a matter of debate. While one study reports no infiltration of lymphocytes in the brain after the ablation of MOG^+^ oligodendrocytes [55]; however, another study shows infiltration of CD4^+^ lymphocytes in the white matter long after the remyelination and causes secondary demyelination and mouse death [49]. We found that in our demyelination model, the infiltrated lymphocytes were mainly CD8^+^ T cells in the white matter of the demyelinated brain at 4 weeks after the 3^rd^ round of oligodendrocyte ablation. The reasons for these discrepancies are unclear. In our study, we found that the OPC responses were enhanced following successive oligodendrocyte ablations, and the proliferation capacity of OPCs and the repopulation of myelinating oligodendrocytes in the corpus callosum is higher than in the cortex. Reactive microglia have been reported to facilitate OPC proliferation and differentiation by phagocytosis of myelin debris and secretion of anti-inflammatory cytokines [56, 57], and T cells also have been reported to promote oligodendrocyte differentiation and remyelination [58]; therefore, the much more pronounced and robust microgliosis and T lymphocyte infiltration in corpus callosum than cortex would provide a potential explanation for the region-heterogenous OPC proliferation and oligodendrocyte regeneration.

Promoting adult OPCs to differentiate into mature, myelin-forming oligodendrocytes could enhance remyelination in MS patients and provide significant clinical benefits. A relatively long “time window” between the onset of prolonged demyelination and spontaneous remyelination in the context of chronic neuroinflammation provides opportunities to evaluate the potential pro-myelinating drugs for clinical MS therapy. By assessing the remyelination efficacy of previously reported compound Benztropine, we show that this model is a necessary addition to other demyelination models, like the EAE model, which is frequently used for the study of the immunopathology of MS.

This repeated oligodendrocyte ablation/demyelination model also offers an excellent opportunity to understand the dynamics of oligodendrocyte lineage cells and remyelination and factors affecting the process in myelinating diseases such as MS. Some studies reported that the number of OPCs is drastically reduced in chronic MS lesions [59, 60] and repeated demyelination-remyelination in MS pathogenesis resulted in remyelination failure has been addressed [61]. It has been hypothesized that repeated demyelination-remyelination could lead to OPC-pool depletion, thus causing remyelination failure [62]. They generated the repeated focal demyelination model by stereotaxically injecting ethidium bromide into the caudal cerebellar peduncles (CCP) three times. Complete remyelination occurred in the CCP area 10 weeks following three rounds of EB injection. The number of PDGFRα mRNA-positive cells returned to the normal control level at the CCP area 10 weeks following one round of EB injection, and repeated demyelination-remyelination did not impair OPC response. These data revealed that OPC-pool depletion and remyelination failure are not induced by successive episodes of focal demyelination in the CNS of rats [62]. Here we show that extensive and repeated ablations of Tmem10^+^ oligodendrocytes cause prolonged demyelination but not exhaustion of OPCs. Taken together, all the results suggest that neither focal demyelination nor widespread injury to mature oligodendrocytes could cause OPCs exhaustion, resulting in remyelination failure.

Although remyelination appeared “complete”, the myelin sheath in our model did not fully recover to the control level over an extended period, which was consistent with other reports [63]. Based on the measurements of the inner and outer diameters of myelinated axons and “G” ratios, we revealed that by 20 weeks after the 3^rd^ round of DT injection, DTR/DT mice exhibited an overall downward trend in the inner and outer diameters of myelinated axons when compared with the controls (Suppl. Fig. 11), suggesting that neither the myelin sheath nor the axon had recovered to the normal control level at this stage. The inner diameter of axons was significantly reduced in the DTR/DT mice, presumably due to the shrinkage of axons of less than or equal to 1μm in diameter (≤1 μm) or 2μm to 3 μm (2–3 μm), leading to a slight drop in the G-ratio. The above results indicate that the G-ratio does not always accurately reflect the thickness of myelin sheath, especially under the condition of axonal shrinkage [7].

In summary, the Tmem10-Cre/DTR/DT mouse model based on repeated ablations of Tmem10^+^ oligodendrocyte presents MS-like pathological features, including prolonged demyelination, axonal injury, and neuroinflammation. In this model, the proliferation and differentiation of OPCs to myelinating oligodendrocytes are accelerated, resulting in spontaneous remyelination. The wide “time window” between the onset of demyelination and spontaneous remyelination makes it suitable for evaluating the efficacy of small molecules for remyelination. Thus, our study could provide a novel insight for MS therapy.

## Methods

All methods were performed in accordance with the relevant guidelines and regulations.

### Mice

The primer sequences used to identify Tmem10-Cre mouse (homemade) by PCR were as follows: sense primer: 5’-CGACCACTACCAGCAGAACA-3’ and antisense primer: 5’-ATGTTTAGCTGGCCCAAATG-3’. tdTomato-reporter mouse (129s6-Gt (ROSA)26tm9(CAG-tdTomato)Hze/J) mouse and Rosa26iDTR mouse (JAX stock #007900) were purchased from the Jax lab, the primer sequences used to identify Rosa26iDTR mice were: forward primer: 5’- AAAGTCGCTCTGAGTTGTTAT-3’ and reverse primer: 5’-AATAGGAACTTCGTCGAGC-3’. Tmem10-Cre mice were crossed with Rosa26iDTR mice to obtain Tmem10-Cre; Rosa26iDTR mice. 6-8-week-old mice were used in our experiments. In order to exclude the effect of DT, all mice were injected with diphtheria toxin (DT, 25 μg/Kg) intraperitoneally. Mice of the proper genotype were allocated randomly into the control and experimental groups and processed alternatively. In this study, n refers to the number of mice and is presented in every figure legend. The sample size was determined by our previous studies related to our experimental assays. All animals were housed under SPF conditions and given a 12h dark/light cycle in a standard environment with sufficient food and water. All animal experiments were carried out strictly according to the Animal Care and Use Committee guidelines of Southern University of Science and Technology (Approval No. SUSTech-JY202110002).

### Western Blotting

Brain tissues, e.g., cortex, were dissected on ice and sonicated in 2% SDS lysis buffer containing protease and phosphatase inhibitors to extract proteins. Generally, 20 μg of total proteins from each sample were loaded into SDS-PAGE gel for electrophoresis and transferred onto 0.22-μm polyvinylidene fluoride membrane (PVDF). The PVDF membranes were blocked with 5% non-fat milk dissolved in TBS containing 1% Tween-20 (TBST) for at least one hour at room temperature and then incubated them with a series of primary antibodies overnight at 4 °C. After being washed three times with TBST, the blots were incubated with secondary antibodies at room temperature for an hour and visualized with the ECL system. The gray values of targeted bands were analyzed by ClinX software. The related primary antibodies were as follows: MOG (Millipore, MAB5680; 1:1000), MOBP (Invitrogen, PA5-72493; 1:1000), MBP (Abcam, ab7349; 1:1000), Tubulin (Biolegend, 801201, 1:20000). The secondary antibodies were as follows: Goat anti-Rabbit IgG (H + L) Secondary Antibody, HRP (Thermo Fisher Scientific, 31460; 1:5000), Goat anti-Mouse IgG (H + L) Secondary Antibody, HRP (Thermo Fisher Scientific, 31430; 1:5000), Anti-rat IgG, HRP-linked Antibody (Cell Signaling Technology, 7077 s; 1:10000).

### Tissue immunofluorescence staining

To prepare brain tissues for sectioning, mice were anesthetized and transcardially perfused with phosphate-buffered saline (PBS) and 4% paraformaldehyde (PFA). The brains were dissected out from these mice and post-fixed for another 24 hours in 4% PFA at 4°C and dehydrated with 20% and 30% sucrose solution in turn. Afterwards, 20-μm brain slices were cut from the tissues embedded with OCT at -20°C for immunostaining. First, slices were blocked with 1% BSA blocking buffer including 0.3% Triton X-100 for an hour at room temperature, then incubated with primary antibodies overnight at 4 °C and washed with PBS three times, before they were incubated with secondary antibodies and DAPI for an hour at room temperature and washed with PBS. The slices were mounted with Fluoromount-G medium (SouthernBiotech, 0100-01) and cover-shipped. The images were captured by Zeiss 800 and analyzed using Zeiss software and Image J software. The primary antibodies were as follows: Sox9 (Oasis, OB-PRB049; 1:200), CC1 (Oasis, OB-PRB070; 1:200), Sox10 (Oasis, OB-PRB001; 1:200), MYRF (Oasis, OB-PRB007; 1:200), PDGFRα (Cell Signaling Technology, 3174S; 1:200), BCAS1 (Santa Cruz Biotechnology, sc-136342; 1:200), GFAP (Millipore, MAB360; 1:500), Iba1 (WAKO, 019-19741; 1:500), CD68 (Abcam, ab53444; 1:500), SMI32 (Biolegend, 801701; 1:500), Ki67 (Invitrogen, 14-5698-80; 1:200), CD3 (Abcam, ab16669; 1:100), CD45R (Abcam, ab64100; 1:200), CD4 (Invitrogen, 14-0041-82; 1:100), CD8α (Invitrogen, 14-0081-82; 1:100). The secondary antibodies were as follows: Goat-anti mouse Alexa Fluor 488 (Thermo Fisher Scientific, A21202; 1:500), Goat-anti rabbit Alexa Fluor 568 (Thermo Fisher Scientific, A10042; 1:500), Goat-anti mouse Alexa Fluor 568 (Thermo Fisher Scientific, A10037; 1:500), Goat-anti rabbit Alexa Fluor 488 (Thermo Fisher Scientific, A21206; 1:500), Goat-anti guinea pig Alexa Fluor guinea pig 488 (Abcam, ab150185; 1:500), Goat-anti rat Alexa Fluor 488 (Thermo Fisher Scientific, A21208; 1:500), Goat-anti rat Alexa Fluor 568 (Thermo Fisher Scientific, A11077; 1:500), DAPI (Sigma, D9542; 1:500).

For quantification of cell numbers, confocal imaging of the cortex and corpus callosum was done with a Zeiss 800 confocal microscope. The comparable area of cortex and corpus callosum between groups of mice were chosen for counting cells of interest with ZEN (Blue edition) software, and cell numbers per mm^2^ were quantified by image J and were normalized to the values of control mice.

### Black Gold staining and Luxol fast blue (LFB) staining

Black Gold staining (Biosensis, TR-100-BG) was performed based on the instructions provided by the manufacturer. Luxol fast blue (LFB) staining was performed based on the protocol provided [64]. 35-μm frozen brain slices were used for Black-Gold staining and LFB staining to examine the content of myelin. The images were captured by Zeiss Axio Imager A2. The intensity per area of black gold staining and LFB staining was analyzed by image J.

### Assessment of the efficacy of Benztropine towards remyelination

To assess the efficacy of Benztropine towards remyelination in the chronic demyelination model generated by repeated oligodendrocyte ablations, Benztropine (Selleck, S3163; 10 mg/Kg) was dissolved in PBS and were i.p. injected into DTR/DT mice once daily for 2 consecutive weeks, DTR/DT mice receiving PBS in the same way were as control. Two weeks right after the end of Benztropine administration, all samples were sacrificed to analyze.

### Electron microscopy (EM)

Samples used for EM were prepared by high-pressure freezing (Leica EM ICE) as described previously [65]. Then the samples were cut into 70-nm ultrathin sections using an Ultramicrotome (Leica EM UC7). The ultrathin sections were stained with 2% uranyl acetate for 18 min and then with lead-citrate for 5 min. EM pictures were captured by a transmission electron microscope (HITACHI, HT7700). The g-ratio of the optic nerve and the corpus callosum was analyzed by ImageJ.

### Statistical analysis

The statistical analysis was done by an independent data analyst. All the statistical analysis was done by GraphPad Prism 8 and Image J. The error bar meant SD or SEM. The different *P* values were depicted as follows: *p < 0.05; **p < 0.01; ***p < 0.001, ****p < 0.0001. Data were analyzed by two-tailed Student’s *t* test, one-way ANOVA or two-way ANOVA.

### Data sharing statement

All research data are available in this article and supplementary materials.

## Supplementary information

Supporting information files

Original data files-Western Blots

## References

[1] Lassmann H. Multiple sclerosis pathology. Cold Spring Harbor Perspect Med. 2018;8. 10.1101/cshperspect.a028936 PMC5830904 29358320

[2] Reich DS, Lucchinetti CF, Calabresi PA. Multiple sclerosis. New Engl J Med. 2018;378:169–80. 29320652 10.1056/NEJMra1401483 PMC6942519

[3] Dobson R, Giovannoni G. Multiple sclerosis—a review. European J Neurol. 2019;26:27–40. 30300457 10.1111/ene.13819

[4] Ineichen BV, Moridi T, Granberg T, Piehl F. Rituximab treatment for multiple sclerosis. Multiple Scler (Houndmills, Basingstoke, Engl). 2020;26:137–52. 10.1177/1352458519858604 31237800

[5] Lassmann H, van Horssen J, Mahad D. Progressive multiple sclerosis: pathology and pathogenesis. Nature Rev Neurol. 2012;8:647–56. 23007702 10.1038/nrneurol.2012.168

[6] Cree BAC, Arnold DL, Chataway J, Chitnis T, Fox RJ, Pozo Ramajo A, et al. Secondary progressive multiple sclerosis: new insights. Neurology. 2021;97:378–88. 34088878 10.1212/WNL.0000000000012323 PMC8397587

[7] Franklin RJ, Ffrench-Constant C. Remyelination in the CNS: from biology to therapy. Nat Rev Neurosci. 2008;9:839–55. 18931697 10.1038/nrn2480

[8] Correale J, Gaitán MI, Ysrraelit MC, Fiol MP. Progressive multiple sclerosis: from pathogenic mechanisms to treatment. Brain: J Neurol. 2017;140:527–46. 10.1093/brain/aww258 27794524

[9] Irvine KA, Blakemore WF. Remyelination protects axons from demyelination-associated axon degeneration. Brain: J Neurol. 2008;131:1464–77. 10.1093/brain/awn080 18490361

[10] Ricigliano VAG, Tonietto M, Hamzaoui M, Poirion É, Lazzarotto A, Bottlaender M, et al. Spontaneous remyelination in lesions protects the integrity of surrounding tissues over time in multiple sclerosis. Eur J Neurol. 2022;29:1719–29. 35152511 10.1111/ene.15285

[11] Kuhlmann T, Miron V, Cui Q, Wegner C, Antel J, Brück W. Differentiation block of oligodendroglial progenitor cells as a cause for remyelination failure in chronic multiple sclerosis. Brain: J Neurol. 2008;131:1749–58. 10.1093/brain/awn096 18515322

[12] Chang A, Tourtellotte WW, Rudick R, Trapp BD. Premyelinating oligodendrocytes in chronic lesions of multiple sclerosis. New Engl J Med. 2002;346:165–73. 11796850 10.1056/NEJMoa010994

[13] Gharagozloo M, Mace JW, Calabresi PA. Animal models to investigate the effects of inflammation on remyelination in multiple sclerosis. Front Mol Neurosci. 2022;15:995477. 36407761 10.3389/fnmol.2022.995477 PMC9669474

[14] Amor S, Groome N, Linington C, Morris MM, Dornmair K, Gardinier MV, et al. Identification of epitopes of myelin oligodendrocyte glycoprotein for the induction of experimental allergic encephalomyelitis in SJL and Biozzi AB/H mice. J Immunol. 1994;153:4349–56. 7525700

[15] Ransohoff RM. Animal models of multiple sclerosis: the good, the bad and the bottom line. Nature Neurosci. 2012;15:1074–7. 22837037 10.1038/nn.3168 PMC7097342

[16] Xing YL, Röth PT, Stratton JA, Chuang BH, Danne J, Ellis SL, et al. Adult neural precursor cells from the subventricular zone contribute significantly to oligodendrocyte regeneration and remyelination. J Neurosci. 2014;34:14128–46. 25319708 10.1523/JNEUROSCI.3491-13.2014 PMC6705285

[17] Golan N, Adamsky K, Kartvelishvily E, Brockschnieder D, Möbius W, Spiegel I, et al. Identification of Tmem10/Opalin as an oligodendrocyte enriched gene using expression profiling combined with genetic cell ablation. Glia. 2008;56:1176–86. 18571792 10.1002/glia.20688 PMC2830273

[18] Jiang W, Yang W, Yang W, Zhang J, Pang D, Gan L, et al. Identification of Tmem10 as a novel late-stage oligodendrocytes marker for detecting hypomyelination. Int J Biol Sci. 2013;10:33–42. 24391449 10.7150/ijbs.7526 PMC3879589

[19] Jäkel S, Agirre E, Mendanha Falcão A, van Bruggen D, Lee KW, Knuesel I, et al. Altered human oligodendrocyte heterogeneity in multiple sclerosis. Nature. 2019;566:543–7. 30747918 10.1038/s41586-019-0903-2 PMC6544546

[20] Yoshikawa F, Sato Y, Tohyama K, Akagi T, Furuse T, Sadakata T, et al. Mammalian-specific central myelin protein opalin is redundant for normal myelination: structural and behavioral assessments. PloS one. 2016;11:e0166732. 27855200 10.1371/journal.pone.0166732 PMC5113975

[21] Buch T, Heppner FL, Tertilt C, Heinen TJ, Kremer M, Wunderlich FT, et al. A Cre-inducible diphtheria toxin receptor mediates cell lineage ablation after toxin administration. Nature Methods. 2005;2:419–26. 15908920 10.1038/nmeth762

[22] Prineas JW, Barnard RO, Kwon EE, Sharer LR, Cho ES. Multiple sclerosis: remyelination of nascent lesions. Annals Neurol. 1993;33:137–51. 10.1002/ana.410330203 8434875

[23] Kuhlbrodt K, Herbarth B, Sock E, Hermans-Borgmeyer I, Wegner M. Sox10, a novel transcriptional modulator in glial cells. J Neurosci. 1998;18:237–50. 9412504 10.1523/JNEUROSCI.18-01-00237.1998 PMC6793382

[24] Emery B, Agalliu D, Cahoy JD, Watkins TA, Dugas JC, Mulinyawe SB, et al. Myelin gene regulatory factor is a critical transcriptional regulator required for CNS myelination. Cell. 2009;138:172–85. 19596243 10.1016/j.cell.2009.04.031 PMC2757090

[25] Koenning M, Jackson S, Hay CM, Faux C, Kilpatrick TJ, Willingham M, et al. Myelin gene regulatory factor is required for maintenance of myelin and mature oligodendrocyte identity in the adult CNS. Journal Neurosci. 2012;32:12528–42. 10.1523/JNEUROSCI.1069-12.2012 PMC3752083 22956843

[26] Ito D, Imai Y, Ohsawa K, Nakajima K, Fukuuchi Y, Kohsaka S. Microglia-specific localisation of a novel calcium binding protein, Iba1. Brain Res Mol Brain Res. 1998;57:1–9. 9630473 10.1016/s0169-328x(98)00040-0

[27] Ohmi K, Greenberg DS, Rajavel KS, Ryazantsev S, Li HH, Neufeld EF. Activated microglia in cortex of mouse models of mucopolysaccharidoses I and IIIB. Proc. Natl Acad Sci USA. 2003;100:1902–7. 12576554 10.1073/pnas.252784899 PMC149931

[28] Ramprasad MP, Terpstra V, Kondratenko N, Quehenberger O, Steinberg D. Cell surface expression of mouse macrosialin and human CD68 and their role as macrophage receptors for oxidized low density lipoprotein. Proc Natl Acad Sci USA. 1996;93:14833–8. 8962141 10.1073/pnas.93.25.14833 PMC26222

[29] Hol EM, Pekny M. Glial fibrillary acidic protein (GFAP) and the astrocyte intermediate filament system in diseases of the central nervous system. Curr Opin Cell Biol. 2015;32:121–30. 25726916 10.1016/j.ceb.2015.02.004

[30] Fransen NL, Hsiao CC, van der Poel M, Engelenburg HJ, Verdaasdonk K, Vincenten MCJ, et al. Tissue-resident memory T cells invade the brain parenchyma in multiple sclerosis white matter lesions. Brain. 2020;143:1714–30. 32400866 10.1093/brain/awaa117

[31] Murphy AC, Lalor SJ, Lynch MA, Mills KH. Infiltration of Th1 and Th17 cells and activation of microglia in the CNS during the course of experimental autoimmune encephalomyelitis. Brain Behav Immun. 2010;24:641–51. 20138983 10.1016/j.bbi.2010.01.014

[32] Machado-Santos J, Saji E, Tröscher AR, Paunovic M, Liblau R, Gabriely G, et al. The compartmentalized inflammatory response in the multiple sclerosis brain is composed of tissue-resident CD8+ T lymphocytes and B cells. Brain. 2018;141:2066–82. 29873694 10.1093/brain/awy151 PMC6022681

[33] van Nierop GP, van Luijn MM, Michels SS, Melief MJ, Janssen M, Langerak AW, et al. Phenotypic and functional characterization of T cells in white matter lesions of multiple sclerosis patients. Acta Neuropathol. 2017;134:383–401. 28624961 10.1007/s00401-017-1744-4 PMC5563341

[34] Traka M, Arasi K, Avila RL, Podojil JR, Christakos A, Miller SD, et al. A genetic mouse model of adult-onset, pervasive central nervous system demyelination with robust remyelination. Brain. 2010;133:3017–29. 20851998 10.1093/brain/awq247 PMC4415057

[35] Pohl HB, Porcheri C, Mueggler T, Bachmann LC, Martino G, Riethmacher D, et al. Genetically induced adult oligodendrocyte cell death is associated with poor myelin clearance, reduced remyelination, and axonal damage. J Neurosci. 2011;31:1069–80. 21248132 10.1523/JNEUROSCI.5035-10.2011 PMC6632929

[36] Criste G, Trapp B, Dutta R. Axonal loss in multiple sclerosis: causes and mechanisms. Handbook Clin Neurol. 2014;122:101–13. 10.1016/B978-0-444-52001-2.00005-4 24507515

[37] Kuhlmann T, Lingfeld G, Bitsch A, Schuchardt J, Brück W. Acute axonal damage in multiple sclerosis is most extensive in early disease stages and decreases over time. Brain. 2002;125:2202–12. 12244078 10.1093/brain/awf235

[38] Trapp BD, Ransohoff RM, Fisher E, Rudick RA. Neurodegeneration in multiple sclerosis: relationship to neurological disability. Neuroscientist. 1999;5:48–57.

[39] Friese MA, Schattling B, Fugger L. Mechanisms of neurodegeneration and axonal dysfunction in multiple sclerosis. Nat Rev Neurol. 2014;10:225–38. 24638138 10.1038/nrneurol.2014.37

[40] Trapp BD, Peterson J, Ransohoff RM, Rudick R, Mörk S, Bö L. Axonal transection in the lesions of multiple sclerosis. New Engl J Med. 1998;338:278–85. 9445407 10.1056/NEJM199801293380502

[41] Franklin RJ. Why does remyelination fail in multiple sclerosis?. Nature Rev Neurosci. 2002;3:705–14. 12209119 10.1038/nrn917

[42] Fard MK, van der Meer F, Sánchez P, Cantuti-Castelvetri L, Mandad S, Jäkel S, et al. BCAS1 expression defines a population of early myelinating oligodendrocytes in multiple sclerosis lesions. Sci Transl Med. 2017;9. 10.1126/scitranslmed.aam7816 PMC7116798 29212715

[43] Deshmukh VA, Tardif V, Lyssiotis CA, Green CC, Kerman B, Kim HJ, et al. A regenerative approach to the treatment of multiple sclerosis. Nature. 2013;502:327–32. 24107995 10.1038/nature12647 PMC4431622

[44] Bible E. Multiple sclerosis: Drug-enhanced remyelination in a multiple sclerosis model. Nat Rev Neurol. 2013;9:660. 24189699 10.1038/nrneurol.2013.225

[45] Wekerle H, Meinl E. Multiple sclerosis: An old drug plays a new trick. Nature. 2013;502:314–5. 24107991 10.1038/nature12694

[46] Mei F, Fancy SPJ, Shen YA, Niu J, Zhao C, Presley B, et al. Micropillar arrays as a high-throughput screening platform for therapeutics in multiple sclerosis. Nat Med. 2014;20:954–60. 24997607 10.1038/nm.3618 PMC4830134

[47] Oluich LJ, Stratton JA, Xing YL, Ng SW, Cate HS, Sah P, et al. Targeted ablation of oligodendrocytes induces axonal pathology independent of overt demyelination. J Neurosci. 2012;32:8317–30. 22699912 10.1523/JNEUROSCI.1053-12.2012 PMC6703635

[48] Ghosh A, Manrique-Hoyos N, Voigt A, Schulz JB, Kreutzfeldt M, Merkler D, et al. Targeted ablation of oligodendrocytes triggers axonal damage. PLoS ONE. 2011;6:e22735. 21818378 10.1371/journal.pone.0022735 PMC3144945

[49] Traka M, Podojil JR, McCarthy DP, Miller SD, Popko B. Oligodendrocyte death results in immune-mediated CNS demyelination. Nat Neurosci. 2016;19:65–74. 26656646 10.1038/nn.4193 PMC4837900

[50] Hagan N, Kane JL, Grover D, Woodworth L, Madore C, Saleh J, et al. CSF1R signaling is a regulator of pathogenesis in progressive MS. Cell Death Dis. 2020;11:904. 33097690 10.1038/s41419-020-03084-7 PMC7584629

[51] Marzan DE, Brügger-Verdon V, West BL, Liddelow S, Samanta J, Salzer JL. Activated microglia drive demyelination via CSF1R signaling. Glia. 2021;69:1583–604. 33620118 10.1002/glia.23980 PMC9250806

[52] Miron VE, Boyd A, Zhao JW, Yuen TJ, Ruckh JM, Shadrach JL, et al. M2 microglia and macrophages drive oligodendrocyte differentiation during CNS remyelination. Nat Neurosci. 2013;16:1211–8. 23872599 10.1038/nn.3469 PMC3977045

[53] Hvilsted Nielsen H, Toft-Hansen H, Lambertsen KL, Owens T, Finsen B. Stimulation of adult oligodendrogenesis by myelin-specific T cells. Am J Pathol. 2011;179:2028–41. 21872562 10.1016/j.ajpath.2011.06.006 PMC3181396

[54] Arnett HA, Mason J, Marino M, Suzuki K, Matsushima GK, Ting JP. TNF alpha promotes proliferation of oligodendrocyte progenitors and remyelination. Nat Neurosci. 2001;4:1116–22. 11600888 10.1038/nn738

[55] Locatelli G, Wörtge S, Buch T, Ingold B, Frommer F, Sobottka B, et al. Primary oligodendrocyte death does not elicit anti-CNS immunity. Nat Neurosci. 2012;15:543–50. 22366759 10.1038/nn.3062

[56] Lloyd AF, Miron VE. The pro-remyelination properties of microglia in the central nervous system. Nat Rev Neurol. 2019;15:447–58. 31256193 10.1038/s41582-019-0184-2

[57] Olah M, Amor S, Brouwer N, Vinet J, Eggen B, Biber K, et al. Identification of a microglia phenotype supportive of remyelination. Glia. 2012;60:306–21. 22072381 10.1002/glia.21266

[58] Bieber AJ, Kerr S, Rodriguez M. Efficient central nervous system remyelination requires T cells. Ann Neurol. 2003;53:680–4. 12731006 10.1002/ana.10578

[59] Wolswijk G. Oligodendrocyte precursor cells in the demyelinated multiple sclerosis spinal cord. Brain. 2002;125:338–49. 11844734 10.1093/brain/awf031

[60] Boyd A, Zhang H, Williams A. Insufficient OPC migration into demyelinated lesions is a cause of poor remyelination in MS and mouse models. Acta Neuropathol. 2013;125:841–59. 23595275 10.1007/s00401-013-1112-y PMC3661931

[61] Prineas JW, Barnard RO, Revesz T, Kwon EE, Sharer L, Cho ES. Multiple sclerosis. Pathology of recurrent lesions. Brain. 1993;116:681–93. 8513397 10.1093/brain/116.3.681

[62] Penderis J, Shields SA, Franklin RJ. Impaired remyelination and depletion of oligodendrocyte progenitors does not occur following repeated episodes of focal demyelination in the rat central nervous system. Brain. 2003;126:1382–91. 12764059 10.1093/brain/awg126

[63] Duncan ID, Marik RL, Broman AT, Heidari M. Thin myelin sheaths as the hallmark of remyelination persist over time and preserve axon function. Proc Natl Acad Sci USA. 2017;114:E9685–91. 29078396 10.1073/pnas.1714183114 PMC5692595

[64] Zou J, Zhou L, Du XX, Ji Y, Xu J, Tian J, et al. Rheb1 is required for mTORC1 and myelination in postnatal brain development. Dev Cell. 2011;20:97–108. 21238928 10.1016/j.devcel.2010.11.020 PMC3056331

[65] Yu Z, Yang Z, Ren G, Wang Y, Luo X, Zhu F, *et al*. GATOR2 complex-mediated amino acid signaling regulates brain myelination. *Proc Natl Acad Sci USA* 2022;119. 10.1073/pnas.2110917119 PMC8784133 35022234

