# Supplementary material for: Repeated ablations of mature Tmem10+ oligodendrocytes recapitulates key pathological features of multiple sclerosis with prolonged demyelination
Source: Cell Death Dis. 2025 Oct 6;16(1):691. doi: 10.1038/s41419-025-07996-0 (PMC12501313; doi:10.1038/s41419-025-07996-0)
Supplement: Supplementary file 1 — Supporting information files [file 41419_2025_7996_MOESM1_ESM.docx]

**Supporting Information for**

Repeated Ablations of Mature Tmem10^+^ Oligodendrocytes Recapitulates Key Pathological Features of Multiple Sclerosis with Prolonged Demyelination

Feiyan Zhu^1^, Haijiao Huang^1^, Yuting Shu^2^, Guoru Ren^1^, Bo Jing^1^, Honglin Tan^2^, Wanxiang Jiang^2^, Yiyuan Cui^2^, Paul F. Worley^3,*^, Bo Xiao^1,^*, Mina Chen^2,*^

* Correspondence

Paul F. Worley

**Email:** pworley1@jhmi.edu

Bo Xiao

**Email:** xiaob@sustech.edu.cn

Mina Chen

**Email:** chenmina2010@scu.edu.cn


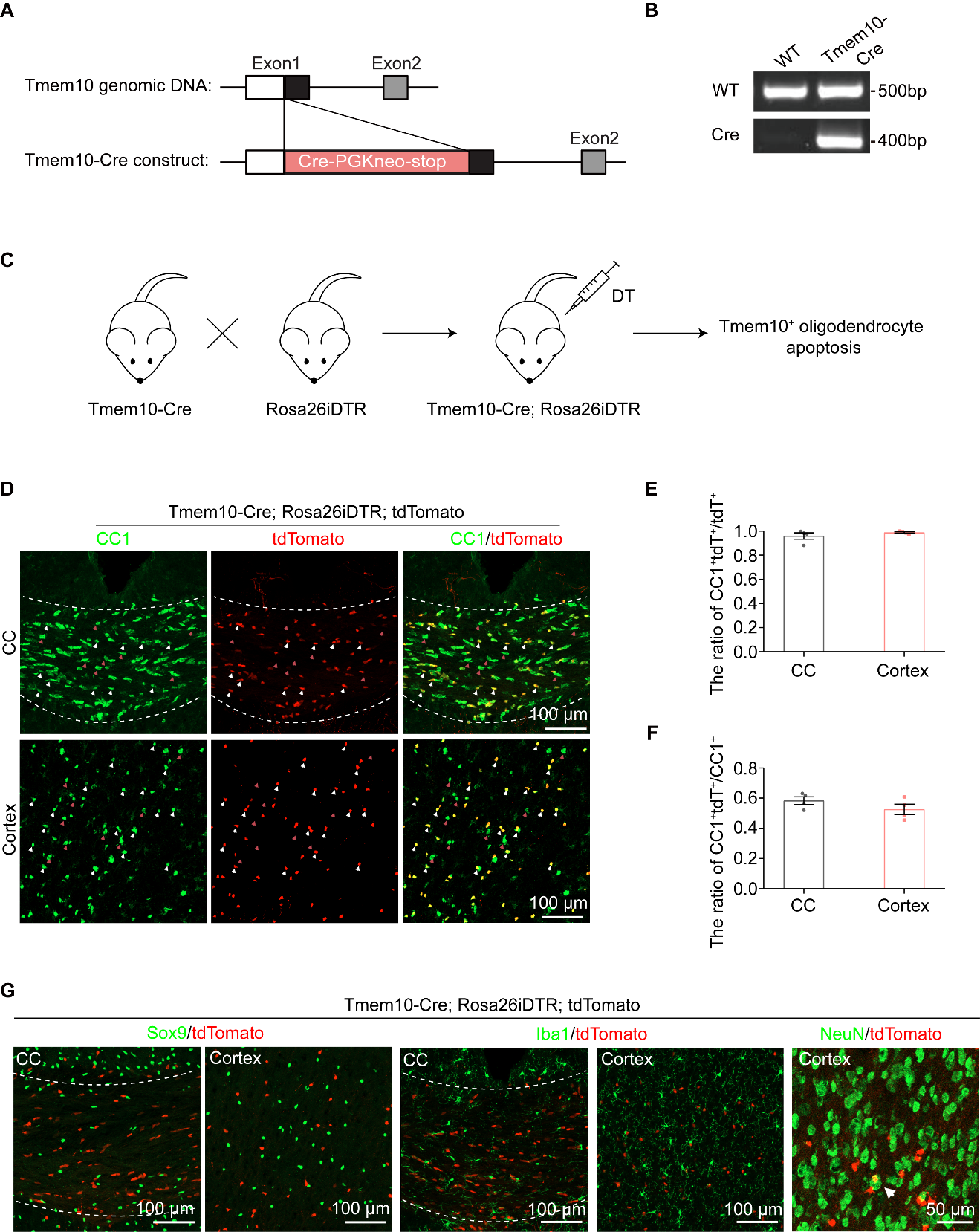
sFig. 1 Construction of Tmem10-Cre; Rosa26iDTR mice.

A. Design illustration for Tmem10-Cre mouse.

B. Genotype validation of Tmem10-Cre mouse by PCR.

C. A brief scheme demonstrating that Tmem10-Cre mouse crossing with Rosa26iDTR mouse rendered Tmem10^+^ oligodendrocytes sensitive to DT.

D-F. tdTomato reporter coupled with CC1 immunostaining and quantification of tdT^+^/CC1^+^ oligodendrocytes in the brain of Tmem10-Cre; Rosa26iDTR; tdTomato mice. The pink arrow heads indicated CC1^+^ single positive cells, and the white arrows indicated tdT^+^/CC1^+^ double positive cells. (n = 4 mice, Age = P30, scale bar = 100μm)

G. tdTomato reporter coupled with Sox9/Iba1/NeuN immunostaining in the brain of Tmem10-Cre; Rosa26iDTR; tdTomato mice. (n = 4 mice per group, Age = P30, scale bar = 100μm except 50μm in NeuN immunostaining)


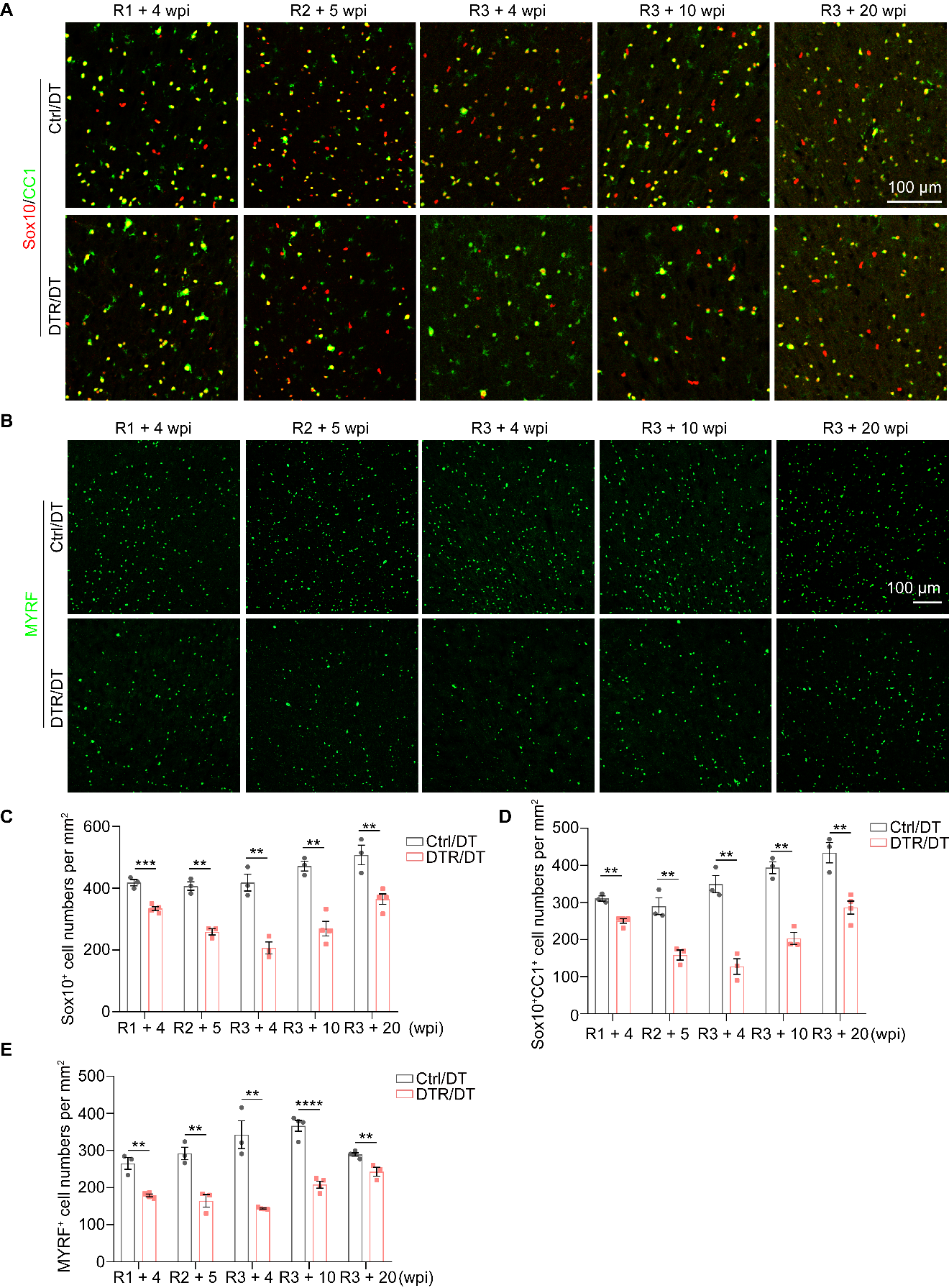


sFig. 2 The dynamics of oligodendrocyte in the cortex of control/DT and DTR/DT mice at different timepoints following repeated DT administrations.

A, C, D. Immunostaining and quantification of Sox10/CC1 showed that the dynamic changes of Sox10^+^ and Sox10^+^CC1^+^ cells in the cortex of DTR/DT mice at different timepoints following three rounds of DT injection. (n = 3-4 mice per group, scale bar = 100μm, mean ± SEM, **p<0.01, ***p<0.001, two-tailed t-test)

B, E. Immunostaining and quantification of MYRF showed that the dynamic changes of MYRF^+^ differentiated oligodendrocytes in the cortex of DTR/DT mice at different timepoints following three rounds of DT injection. (n = 3-4 mice per group, scale bar = 200μm, mean ± SEM, **p<0.01, ****p<0.0001, two-tailed t-test)


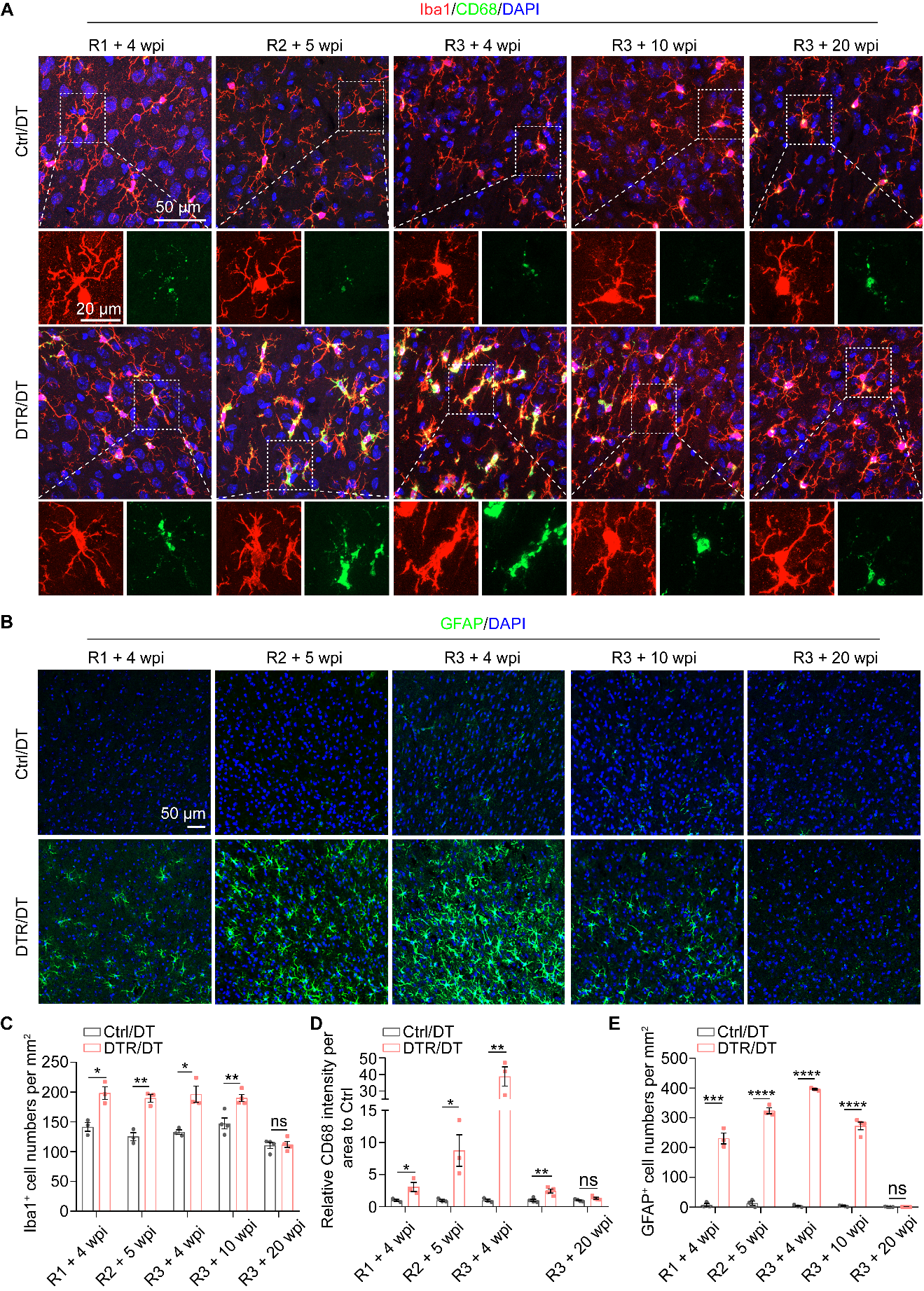
sFig. 3 Chronic neuroinflammation in the cortex after repeated oligodendrocyte ablations.

A, C, D. Iba1/CD68 immunostaining at different timepoints following repeated oligodendrocyte ablations demonstrated that microglia maintained reactive state in cortex once activated by oligodendrocyte death, revealed by increased numbers of Iba1^+^ microglia (C) and CD68 intensity (D), finally returned to normal control level at 20 weeks after the 3^rd^ round of DT injection. (n = 3-4 mice per group, scale bar = 50μm, mean ± SEM, *p<0.05, **p<0.01, not significant, two-tailed t-test). The white box was an enlarged graph, the morphology changes of microglia could be assessed. (scale bar = 20μm)

B, E. Confocal images and quantification of GFAP^+^ astrocytes in the cortex at different timepoints following repeated oligodendrocyte ablations suggested that astrocytes sustained reactive state once activated by 4 weeks after one round oligodendrocyte ablation, finally returned to control level at 20 weeks after the 3^rd^ round of DT injection. (n=3-4 mice per group, scale bar = 50μm, mean ± SEM, ***p<0.001, ****p<0.0001, ns, not significant, two-tailed t-test)


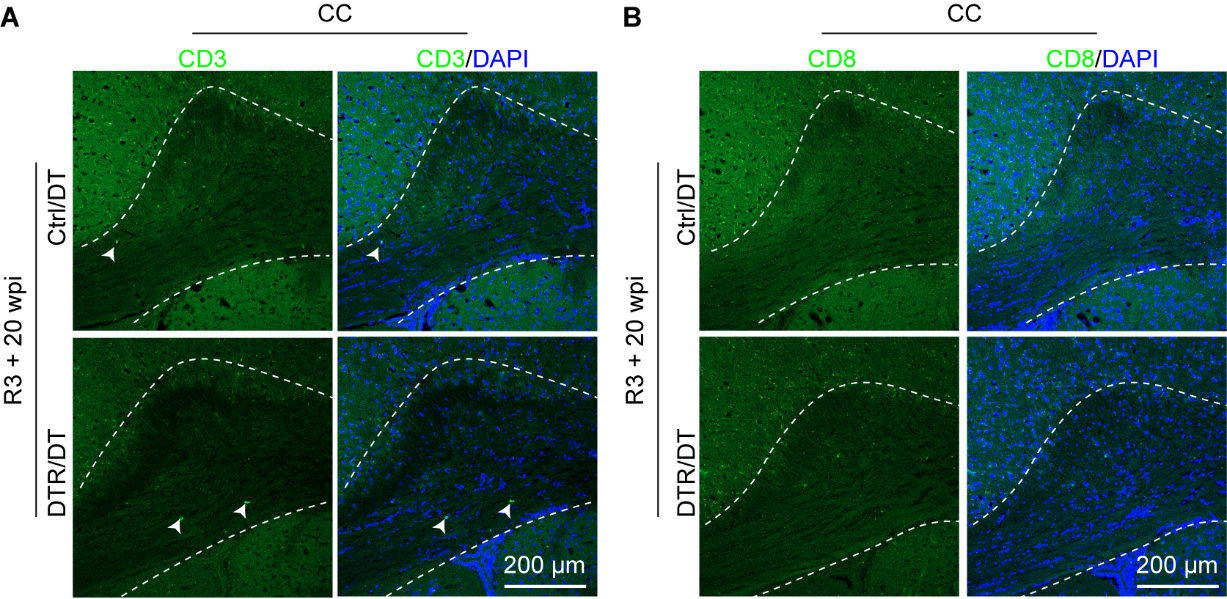


sFig. 4 T lymphocytes disappeared at 20 weeks after the 3^rd^ round of DT injection.

A. CD3 immunostaining indicated that few infiltrated CD3^+^ T immune cells in the corpus callosum of DTR/DT mice at 20 weeks after the 3^rd^ round of DT injection. (n = 4 mice per group, scale bar = 200μm)

B. CD8 immunostaining indicated that nearly no infiltrated CD8^+^ T immune cells in the corpus callosum of DTR/DT mice at 20 weeks after the 3^rd^ round of DT injection. (n = 4 mice per group, scale bar = 200μm)


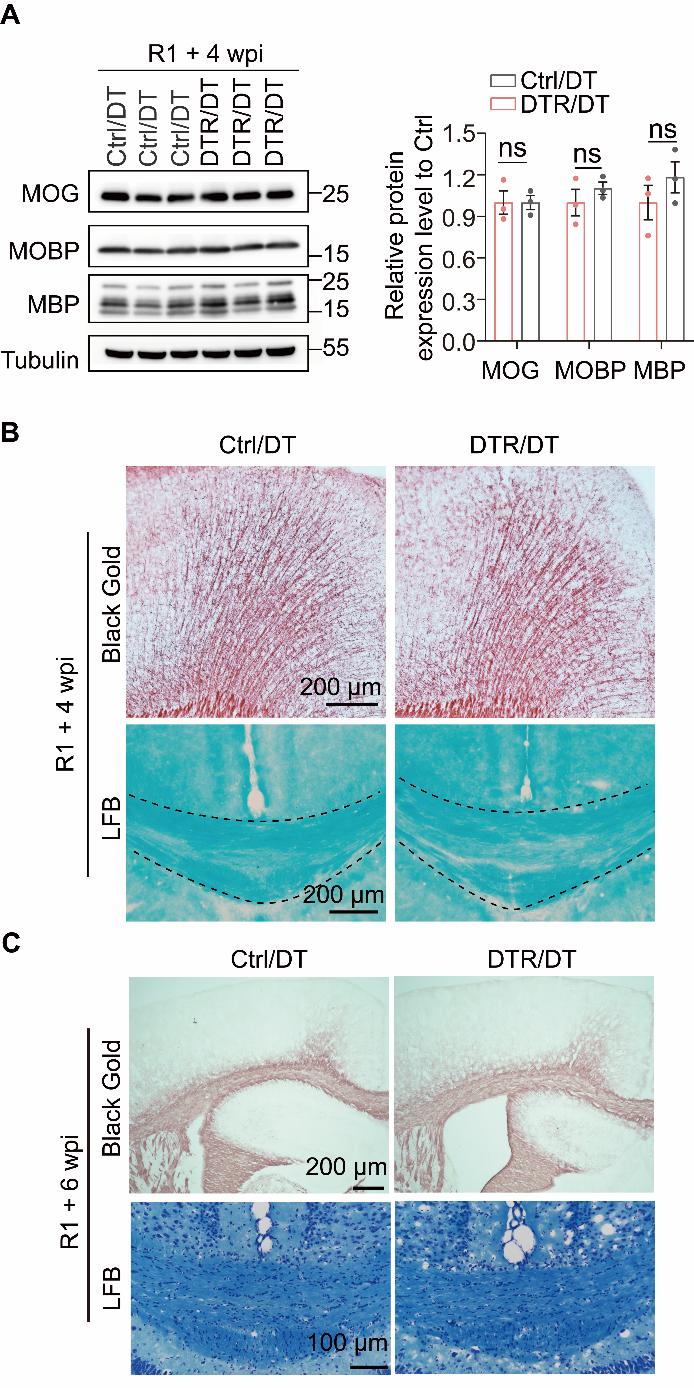


sFig. 5 One round of DT injection to 6-8-week-old DTR mice is not sufficient to trigger demyelination.

A. Western blots and quantification indicated no reduction of myelin related proteins in the cortex of DTR/DT mice compared with control/DT mice at 4 weeks after one round of DT injection. (n=3 mice per group, ns, not significant, two-tailed t-test)

B. Black Gold staining and LFB staining showed myelin content had no change between DTR/DT and control/DT mice at 4 weeks after one round of DT injection. (n=3 mice per group, scale bar = 200μm)

C. Black Gold staining and LFB staining showed myelin content had no change between DTR/DT and control/DT mice at 6 weeks after one round of DT injection. (n=4 mice per group, scale bar = 200μm in Black Gold/100μm in LFB)


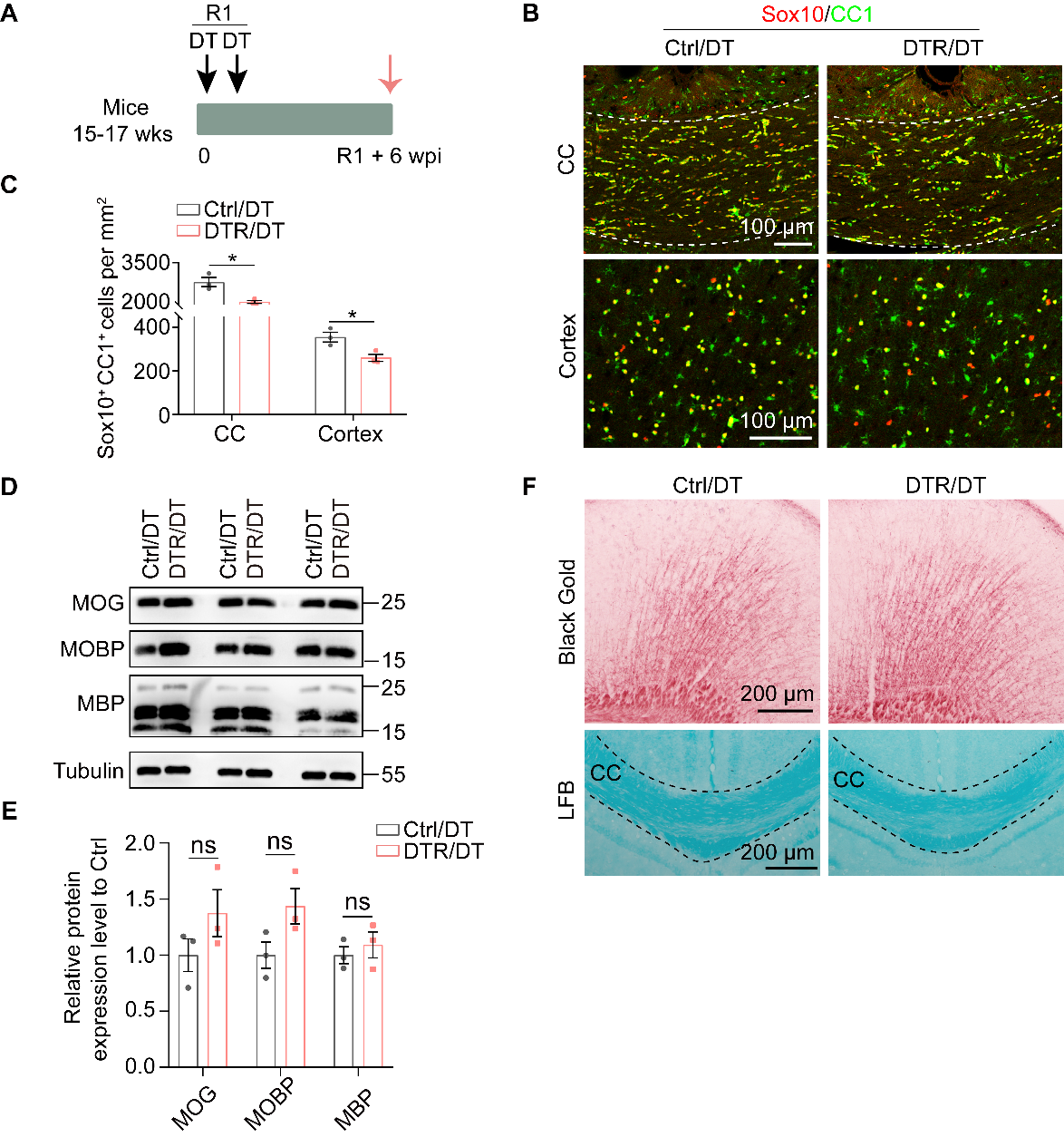


sFig. 6 One round of DT injection to 15-17-week-old DTR mice did not result in demyelination.

A. Schedule of one round of DT treatment (black arrows) to 15-17-week-old DTR mice and timepoints of animals harvested (pink arrows).

B, C. Immunostaining and quantification of Sox10/CC1 showing the number of mature oligodendrocytes decrease to 75% in the corpus callosum and 73% in the cortex of DTR/DT mice, compared with control/DT mice. (n=3 mice per group, scale bar = 100μm, mean ± SEM, *p<0.05, two-tailed t-test)

D, E. Western blots and quantification indicated no reduction of myelin related proteins in the cortex of DTR/DT mice compared with control/DT mice at 6 weeks after one round of DT injection. (n=3 mice per group, mean ± SEM, ns, not significant, two-tailed t-test)

F. Black Gold staining and LFB staining showed myelin had no change between DTR/DT and control/DT mice at 6 weeks after one round of DT injection. (n=3 mice per group, scale bar = 200μm)


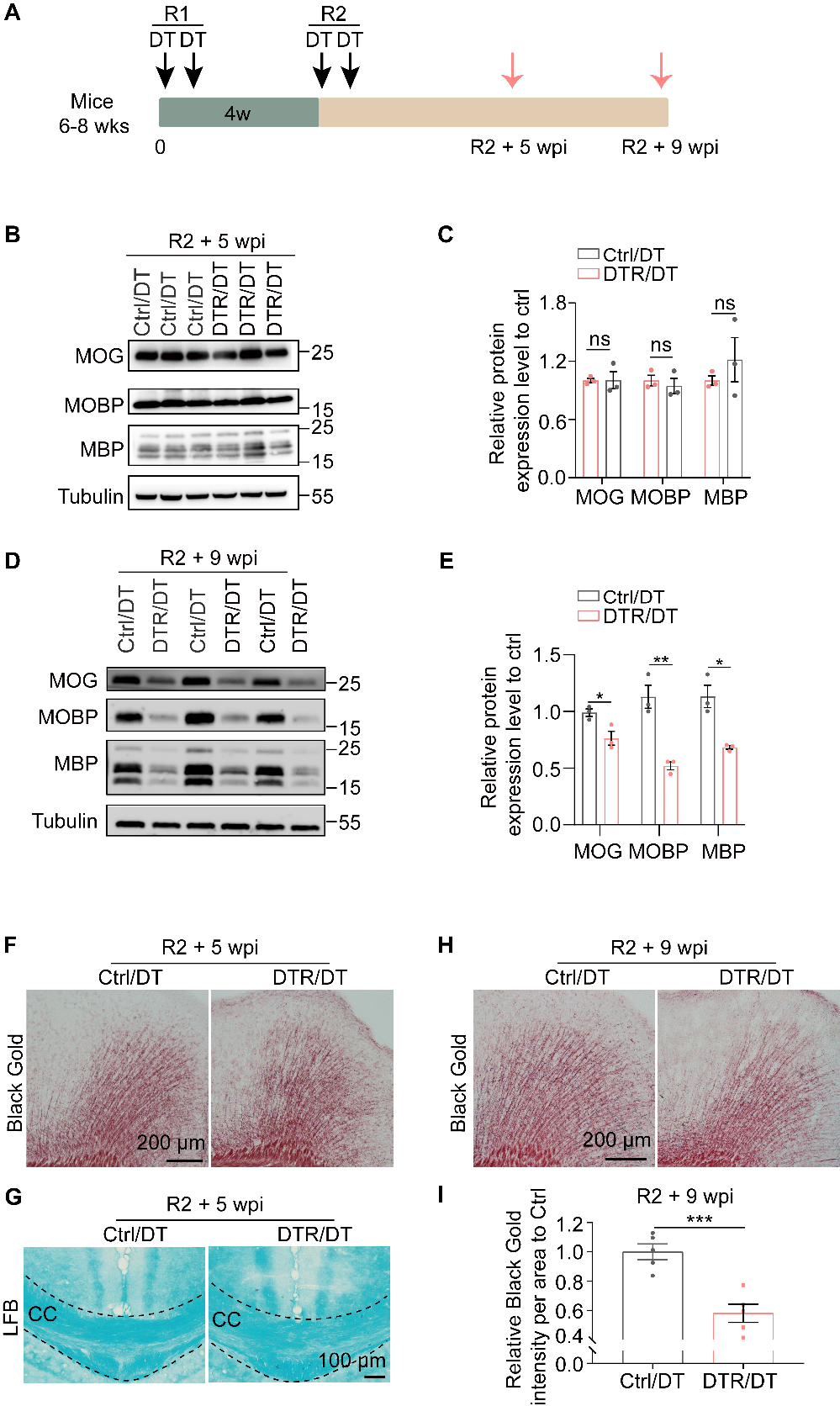


sFig. 7 Two rounds of DT injection to 6-8-week-old DTR mice induced modest demyelination.

A. Schedule of two rounds of DT treatment (black arrows) to 6-8-week-old DTR mice and timepoints of animals harvested (pink arrows).

B, C. Western blots and quantification indicated no significant reduction of myelin related proteins in the cortex of DTR/DT mice compared with control/DT mice at 5 weeks after the 2^nd^ round of DT injection. (n=3 mice per group, mean ± SEM, ns, not significant, two-tailed t-test)

D, E. Western blots and quantification indicated significant reduction of myelin related proteins in the cortex of DTR/DT mice compared with control/DT mice at 9 weeks after the 2^nd^ round of DT injection. (n=3 mice per group, mean ± SEM, *p<0.05, **p<0.01, two-tailed t-test)

F. Black Gold staining showed the intensity of Black Gold had no marked change between DTR/DT mice and control/DT mice at 5 weeks after the 2^nd^ round of DT injection. (n=3 mice per group, scale bar = 200μm)

G. LFB staining showed the intensity of LFB had no marked change between DTR/DT mice and control/DT mice at 5 weeks after the 2^nd^ round of DT injection. (n=3 mice per group, scale bar = 100μm)

H, I. Black Gold staining showed the intensity of Black Glod in DTR/DT mice reduced to 58% of normal control level at 9 weeks after the 2^nd^ round of DT injection. (n=3 mice per group, scale bar = 200μm, mean ± SEM, ***p<0.001, two-tailed t-test)


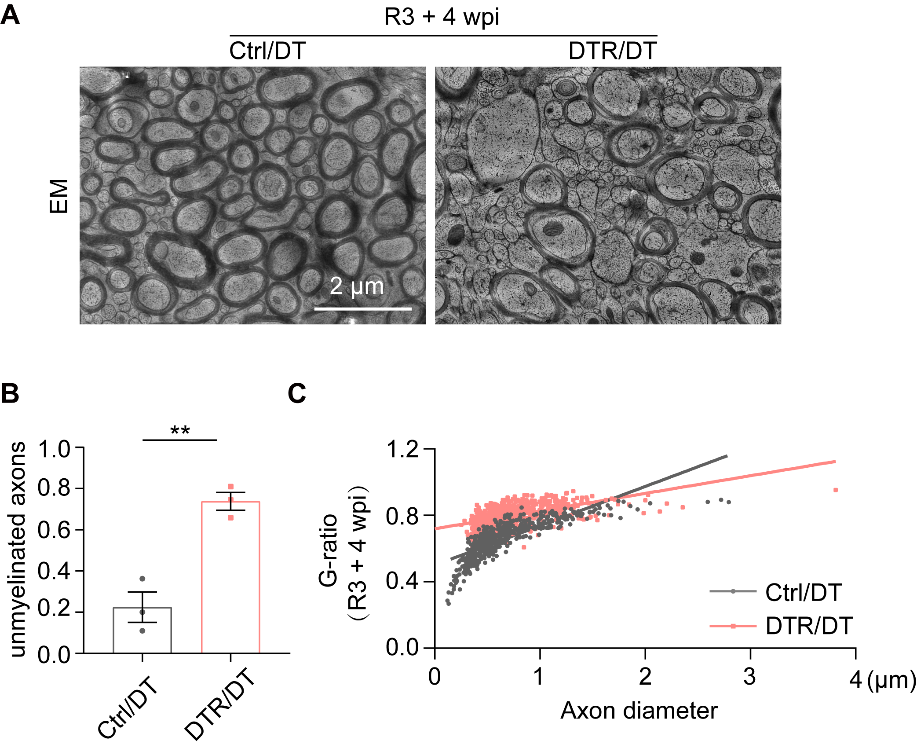


sFig. 8 Three rounds of oligodendrocyte ablations induced obvious demyelination occurred in the corpus callosum of DTR/DT mice.

A-C. Representative images of electron microscopy of corpus callosum and quantification of G-ratio and ratio of unmyelinated axons in control/DT and DTR/DT mice at 4 weeks after the 3^rd^ round of DT injection. (n=3 mice per group, scale bar = 2μm, mean ± SEM, **p<0.01, two-tailed t-test)


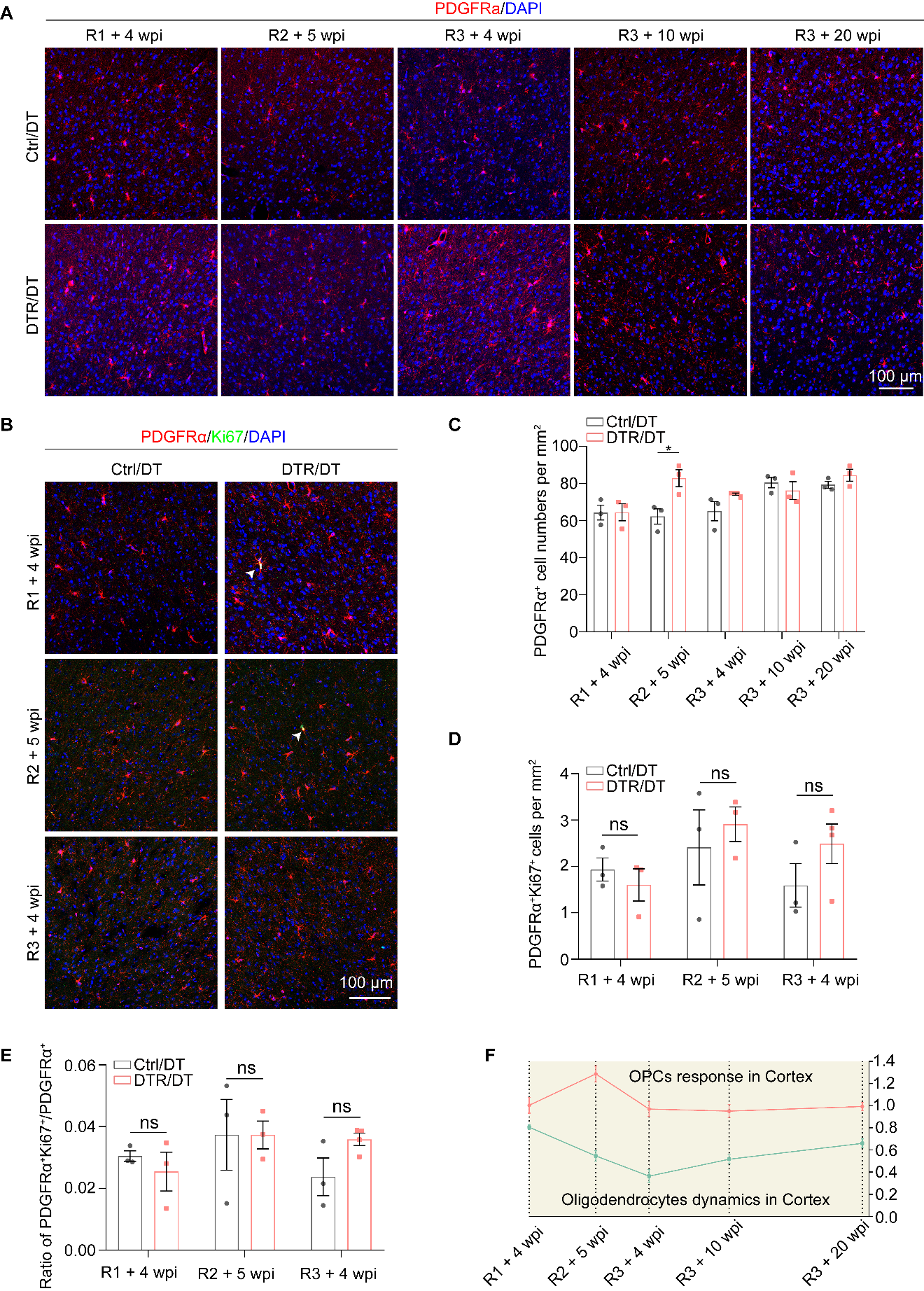
sFig. 9 Proliferation of OPCs in the cortex of DTR/DT mice following repeated oligodendrocyte ablations.

A, C. Immunostaining and quantification of PDGFRα^+^ cells revealed the increased numbers of OPCs in the cortex of DTR/DT mice were only detected at 5 weeks after the 2^nd^ round of DT injection, then the number of OPCs reduced to normal control level at 4 weeks after the 3^rd^ round of DT injection (n=3-4 mice per group, scale bar = 100μm, mean ± SEM, *p<0.05, ns, not significant, two-tailed t-test)

B, D. PDGFRα/Ki67 immunostaining and quantification of the number of OPCs expressing Ki67 in the cortex of DTR/DT and control/DT mice at different timepoints following repeated oligodendrocyte ablations. (n=3-4 mice per group, scale bar = 100μm, mean ± SEM, ns, not significant, two-tailed t-test)

E. Quantification of the ratio of PDGFRα^+^Ki67^+^/PDGFRα^+^ in the cortex of DTR/DT mice and control/DT mice at different timepoints following repeated oligodendrocyte ablations. (n=3-4 mice per group, scale bar = 100μm, mean ± SEM, ns, not significant, two-tailed t-test)

F. A brief diagram demonstrating that the OPC responses were correlated with the repopulation of differentiating oligodendrocytes in the cortex of DTR/DT mice.


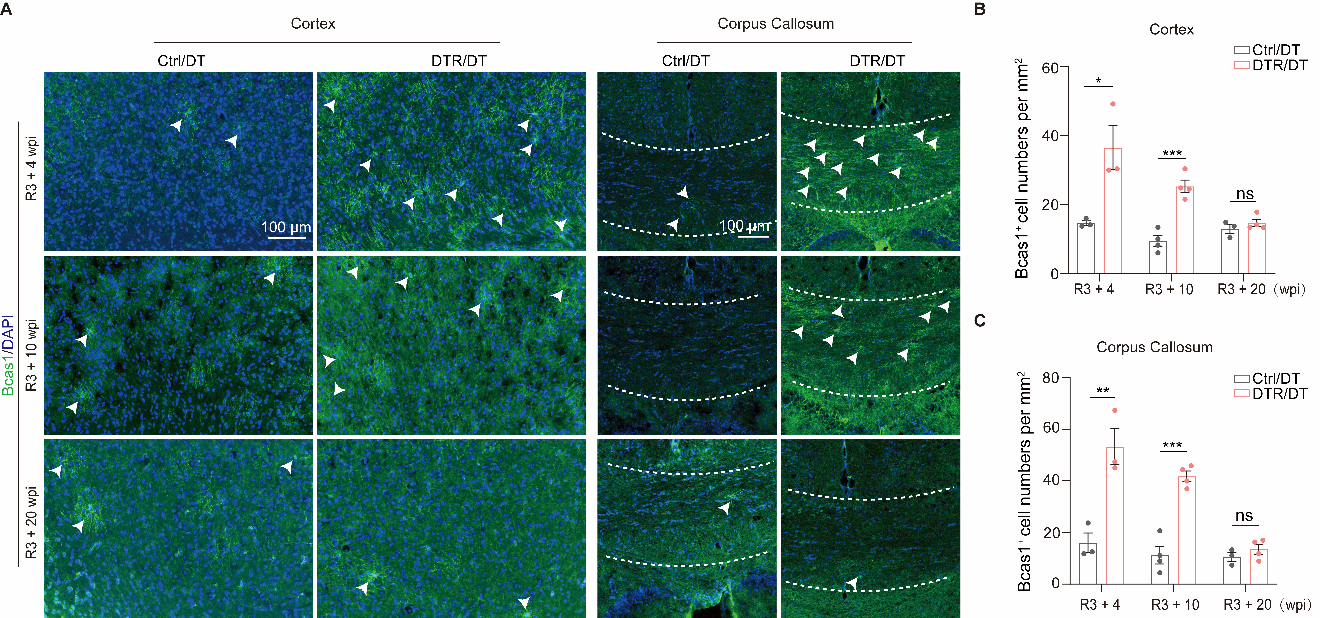


sFig. 10 Differentiation of OPCs in the cortex and corpus callosum of DTR mice following repeated oligodendrocyte ablations.

Immunostaining and quantification of Bcas1 revealed the numbers of Bcas1^+^ cells were increased in both the cortex and corpus callosum (compared with Ctrl/DT mice) of DTR/DT mice at 4 weeks and 10 weeks after the 3^rd^ round of DT injection. At 20 weeks after the 3^rd^ round of DT injection, the number of Bcas1^+^ cells in the cortex and corpus callosum of DTR/DT mice was comparable to that in Ctrl/DT mice. (n=3-4 mice per group, scale bar = 100μm，mean ± SEM, *p<0.05, **p<0.01, ***p<0.001, ns, not significant, two-tailed t-test)


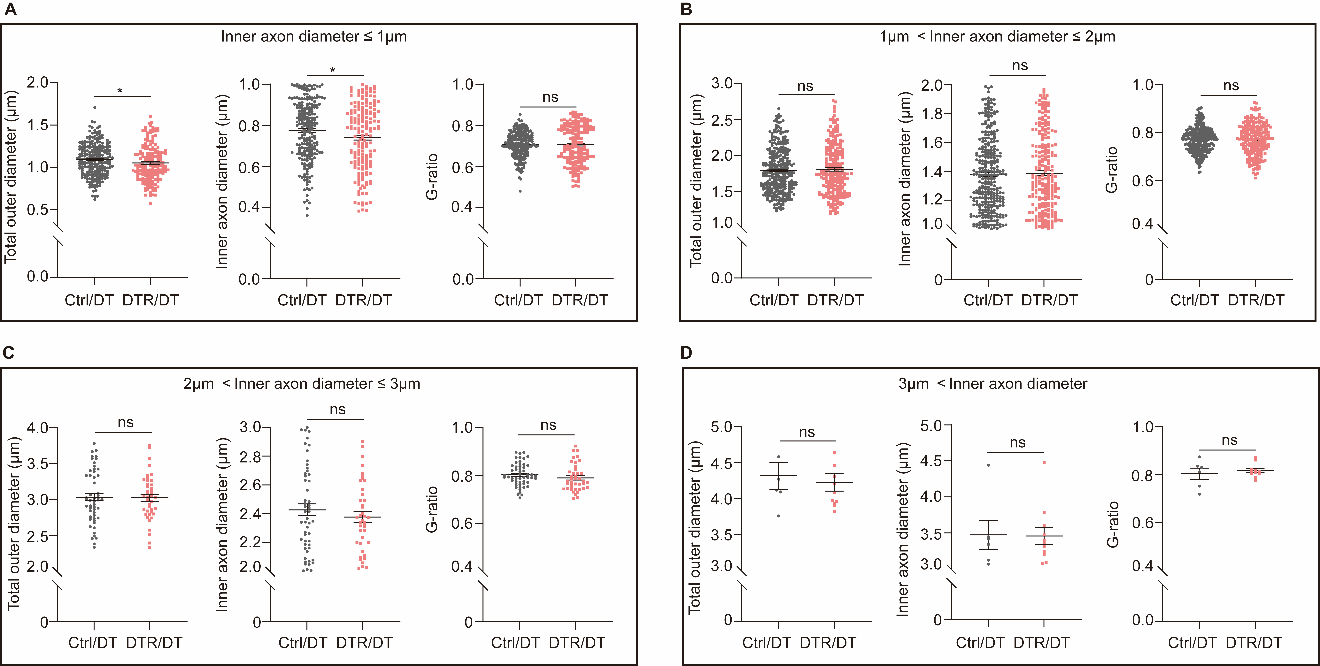


sFig.11 The total outer and inner axonal diameter and related G-ratio of optic nerve of DTR/DT and Ctrl/DT mice at 20 weeks after the 3^rd^ round of DT injection

A. Quantification of the total outer and inner axonal diameter (axon diameter ≤ 1μm) and related G-ratio of optic nerve of DTR/DT and Ctrl/DT mice at 20 weeks after the 3^rd^ round of DT injection. (n = 3-4 mice per group, mean ± SEM, *p<0.05, ns, not significant, two-tailed t-test)

B. Quantification of the total outer and inner axonal diameter (1μm < axon diameter ≤ 2μm) and related G-ratio of optic nerve of DTR/DT and Ctrl/DT mice at 20 weeks after the 3^rd^ round of DT injection. (n = 3-4 mice per group, mean ± SEM, ns, not significant, two-tailed t-test)

C. Quantification of the total outer and inner axonal diameter (2μm < axon diameter ≤ 3μm) and related G-ratio of optic nerve of DTR/DT and Ctrl/DT mice at 20 weeks after the 3^rd^ round of DT injection. (n = 3-4 mice per group, mean ± SEM, ns, not significant, two-tailed t-test)

D. Quantification of the total outer and inner axonal diameter (axon diameter > 3μm) and related G-ratio of optic nerve of DTR/DT and Ctrl/DT mice at 20 weeks after the 3^rd^ round of DT injection. (n = 3-4 mice per group, mean ± SEM, ns, not significant, two-tailed t-test)
