# Supplementary material for: Repeated ablations of mature Tmem10+ oligodendrocytes recapitulates key pathological features of multiple sclerosis with prolonged demyelination
Source: Cell Death Dis. 2025 Oct 6;16(1):691. doi: 10.1038/s41419-025-07996-0 (PMC12501313; doi:10.1038/s41419-025-07996-0)
Supplement: Supplementary file 2 — Original data files-Western Blots [file 41419_2025_7996_MOESM2_ESM.pptx]

## Slide 1
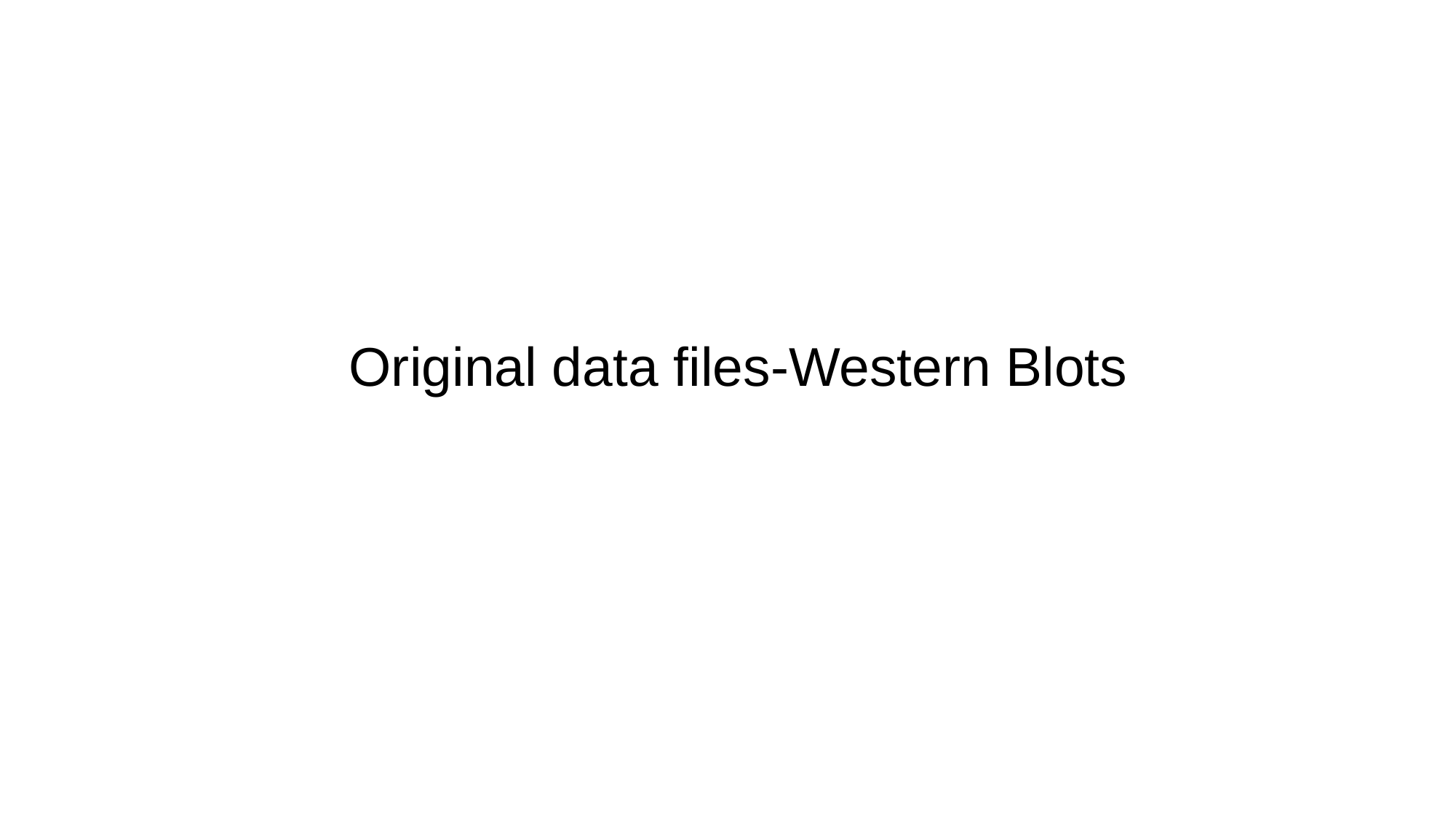

Original data files-Western Blots

## Slide 2
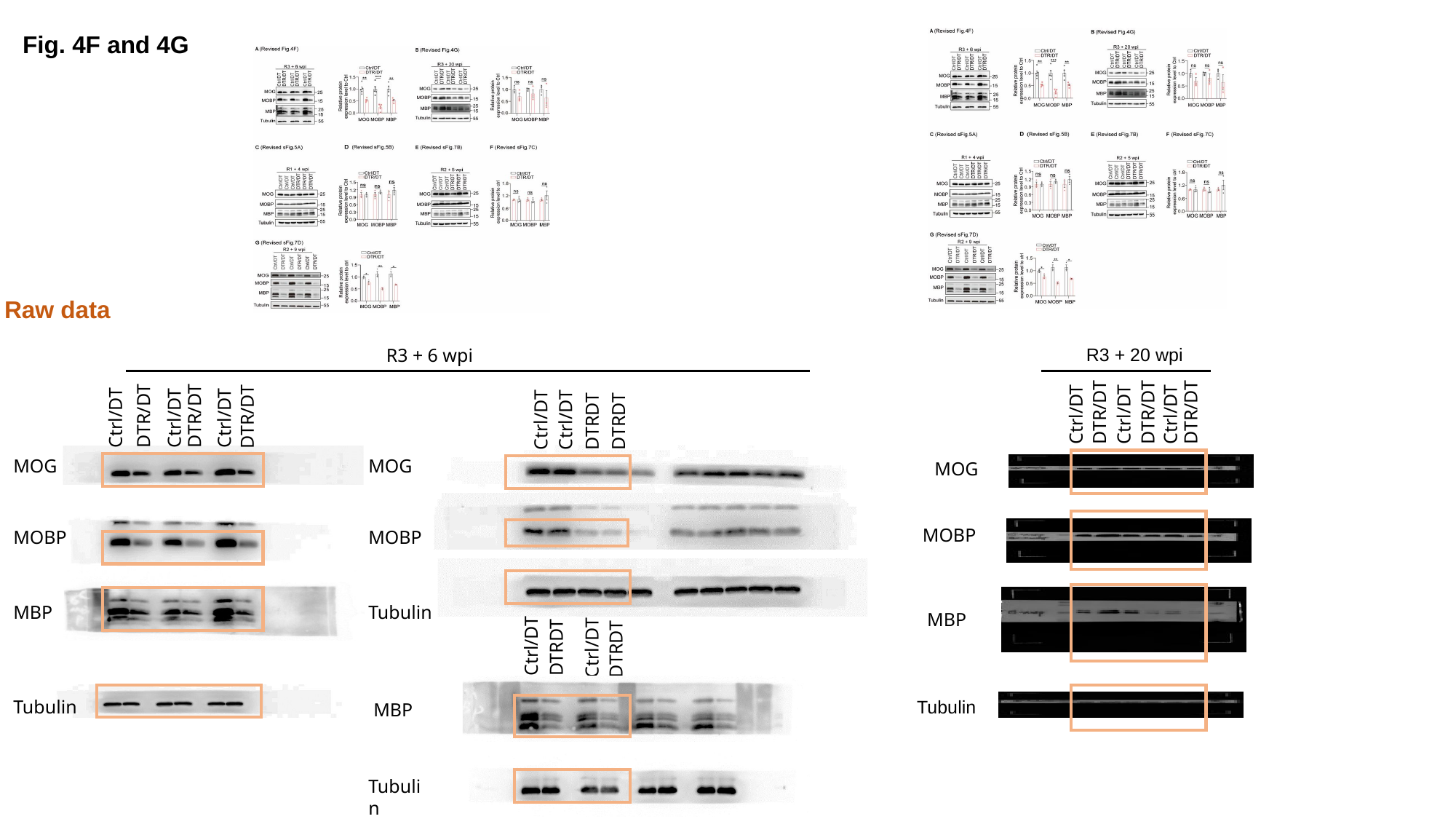

Fig. 4F and 4G
Raw data
R3 + 20 wpi
R3 + 6 wpi
Ctrl/DT
DTR/DT
Ctrl/DT
Ctrl/DT
DTR/DT
Ctrl/DT
DTR/DT
Ctrl/DT
DTR/DT
Ctrl/DT
DTR/DT
DTRDT
DTRDT
Ctrl/DT
Ctrl/DT
DTR/DT
MOG
MOG
MOG
MOBP
MOBP
MOBP
DTRDT
MBP
Tubulin
DTRDT
MBP
Ctrl/DT
Ctrl/DT
Tubulin
Tubulin
MBP
Tubulin

## Slide 3
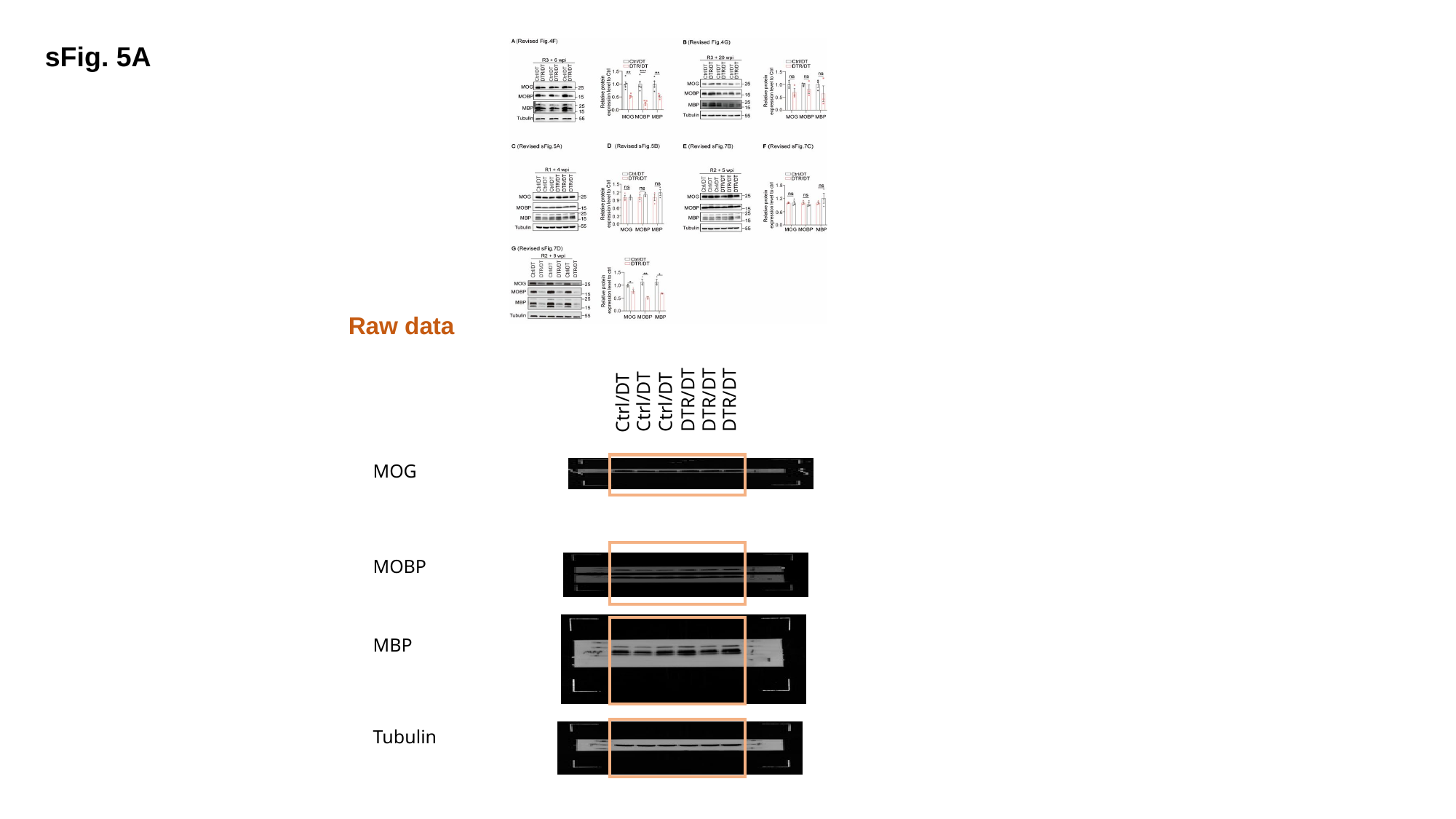

sFig. 5A
Raw data
Ctrl/DT
Ctrl/DT
DTR/DT
DTR/DT
DTR/DT
Ctrl/DT
MOG
MOBP
MBP
Tubulin

## Slide 4
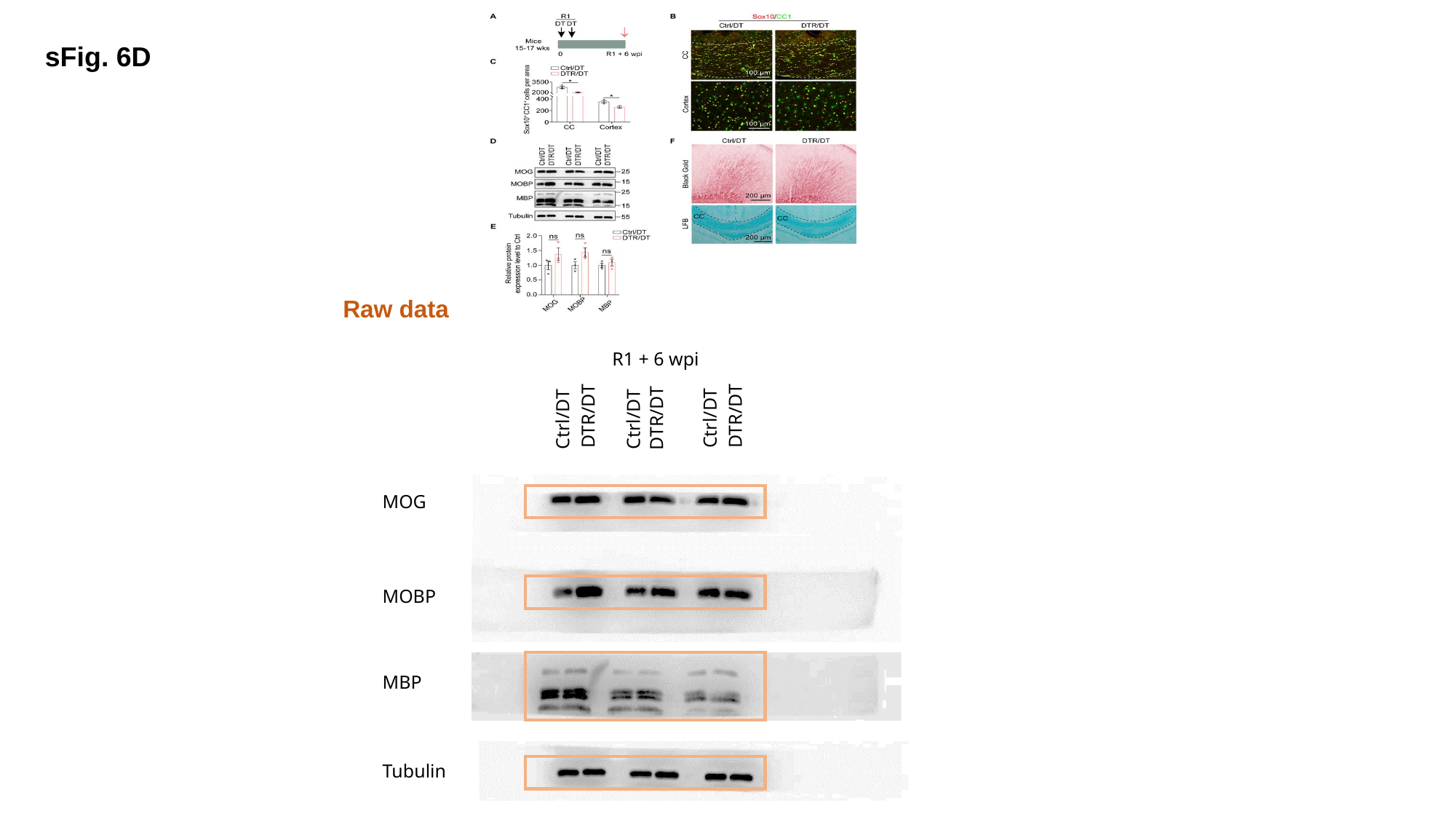

sFig. 6D
Raw data
DTR/DT
Ctrl/DT
DTR/DT
Ctrl/DT
Ctrl/DT
DTR/DT
R1 + 6 wpi
MOG
MOBP
MBP
Tubulin

## Slide 5
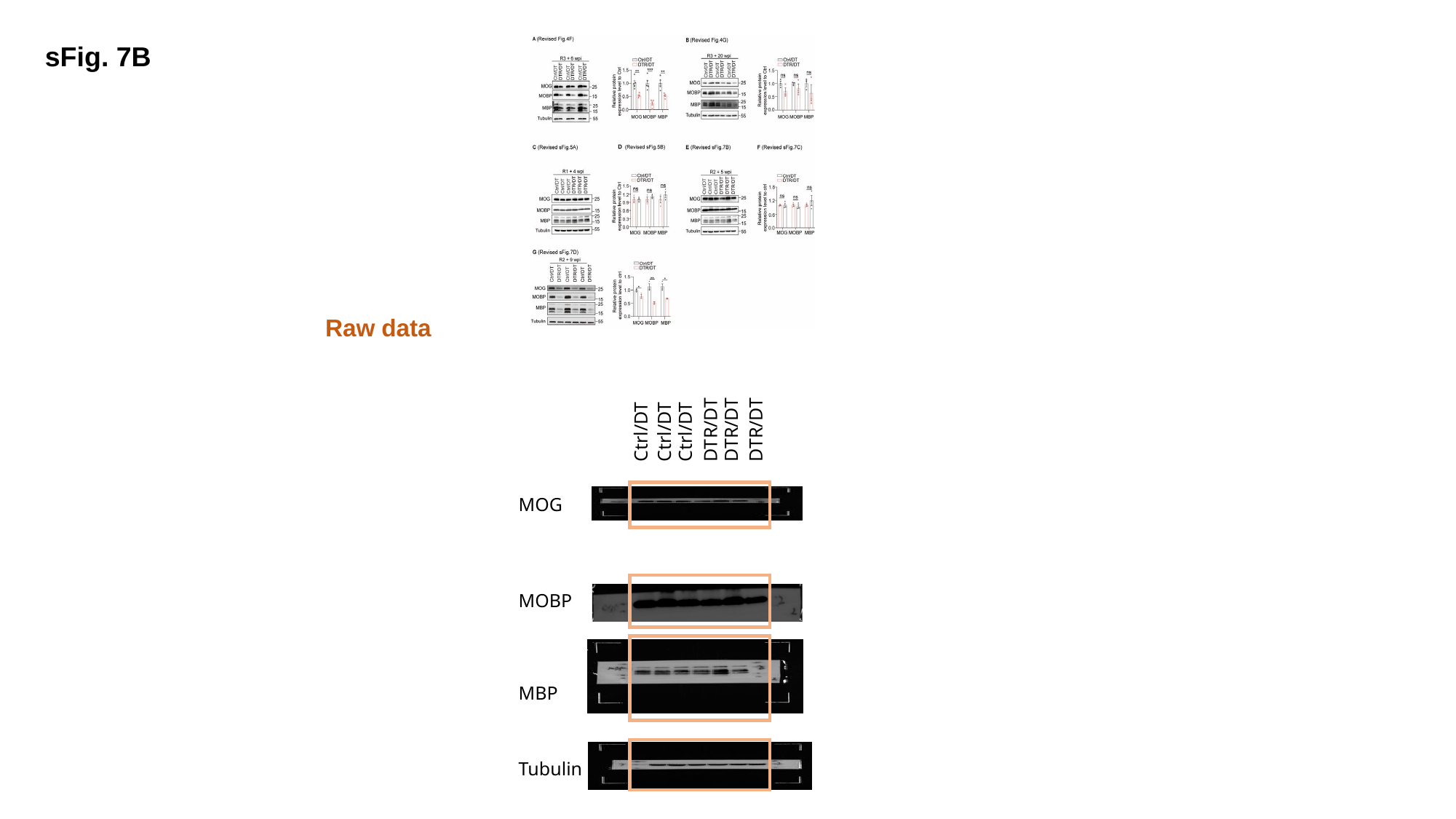

sFig. 7B
Raw data
Ctrl/DT
Ctrl/DT
Ctrl/DT
DTR/DT
DTR/DT
DTR/DT
MOG
MOBP
MBP
Tubulin

## Slide 6
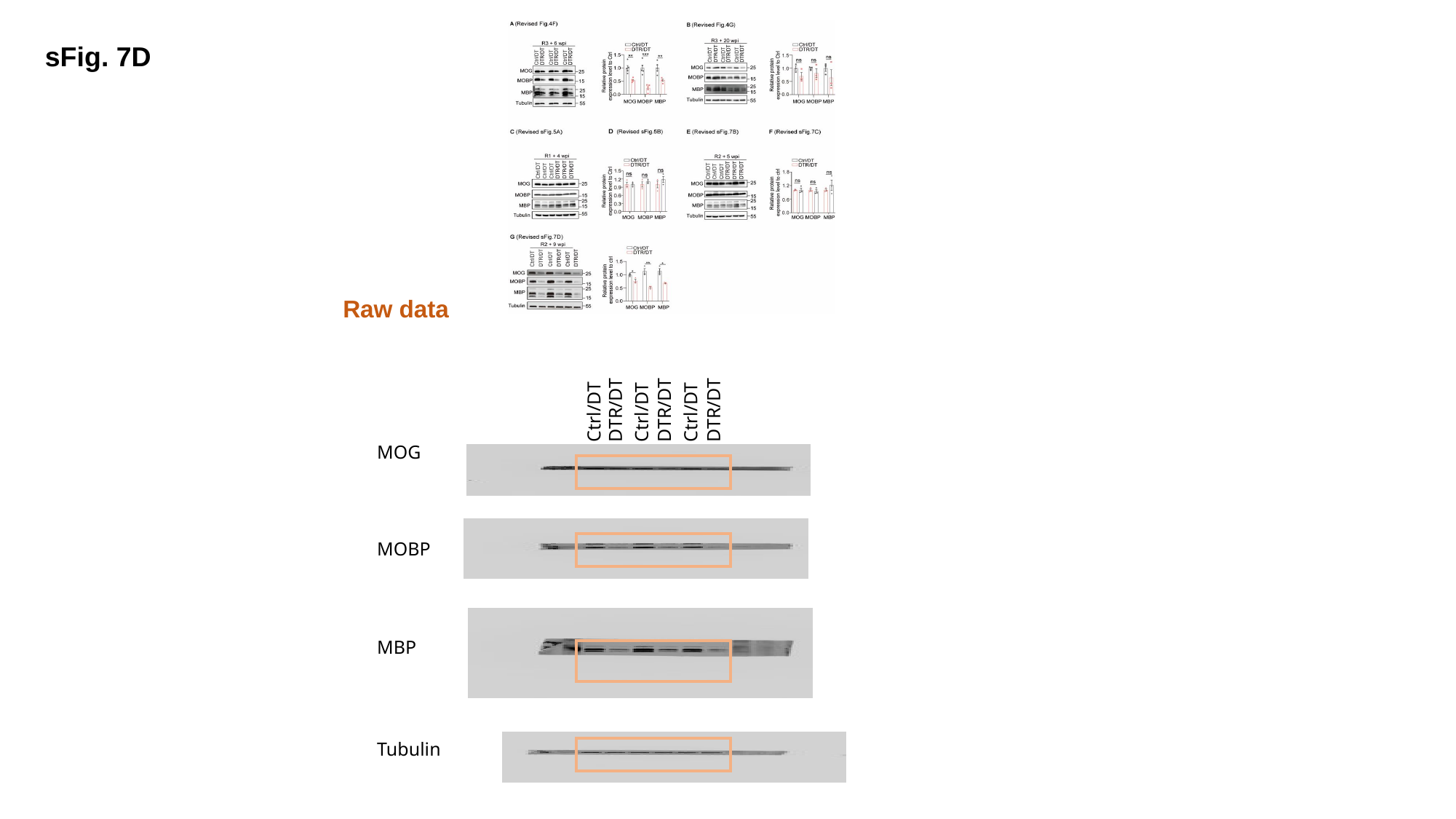

sFig. 7D
Ctrl/DT
DTR/DT
Ctrl/DT
DTR/DT
Ctrl/DT
DTR/DT
Raw data
MOG
MOBP
MBP
Tubulin
